# Stabilized Fe_7_C_3_ catalyst with K–Mg dual promotion for robust CO_2_ hydrogenation to high-value olefins

**DOI:** 10.1038/s41467-025-63218-3

**Published:** 2025-08-28

**Authors:** Fei Qian, Maolin Wang, Zidu Wei, Yi Cai, Zeping Sun, Ruikang Liang, Guangbo Liu, Ming Qing, Hong Wang, Jinjia Liu, Xing-Wu Liu, Yong Yang, Xiao-Dong Wen

**Affiliations:** 1 State Key Laboratory of Coal Conversion, Institute of Coal Chemistry, Chinese Academy of Sciences, Taiyuan, 030001 China; 2 National Energy Center for Coal to Liquids, Synfuels China Co. Ltd., Huairou District Beijing, 101400 China; 3 University of Chinese Academy of Sciences, No. 19A Yuquan Road, Beijing, 100049 PR China; 4 Beijing National Laboratory for Molecular Sciences, New Cornerstone Science Laboratory, College of Chemistry and Molecular Engineering, Peking University, Beijing, China; 5 Key Laboratory of Photoelectric Conversion and Utilization of Solar Energy, Qingdao Institute of Bioenergy and Bioprocess Technology, Chinese Academy of Sciences, Qingdao, 266101 China

**Keywords:** Heterogeneous catalysis, Catalyst synthesis, Chemical engineering

## Abstract

Iron carbide catalysts, particularly the Fe_7_C_3_ phase, hold significant potential for efficient CO_2_ hydrogenation to olefins, yet stabilizing this phase under reactive conditions remains a major challenge. Herein, we report a robust and efficient synthesis of nearly phase-pure Fe_7_C_3_ catalysts derived from Prussian blue analogues, whose stability is significantly enhanced by strategically incorporating K and Mg promoters. Comprehensive characterization reveals that K accelerates the carbonization process and markedly enhances olefin selectivity, whereas Mg effectively suppresses water-induced oxidation, preserving the structural integrity of the Fe_7_C_3_ phase. Under optimized reaction conditions (340 °C, 2 MPa, H_2_/CO_2_ = 3), the Fe_7_C_3_-KMg catalyst achieves a high CO_2_ conversion of 41.5% and an olefin selectivity of 67.1%, maintaining exceptional catalytic stability for over 1000 hours. These findings offer valuable new insights into the rational design of robust iron carbide catalysts for sustainable and efficient CO_2_ conversion into high-value chemicals.

## Abstract (web summary)

Iron carbide catalysts—especially the Fe_7_C_3_; phase—show great promise for efficient CO_2_ hydrogenation to olefins. Here, the authors report the first stable, nearly pure Fe_7_C_3_ catalyst for CO_2_-to-olefins conversion, overturning conventional models that posit the necessity of Fe_5_C_2_–Fe_3_O_4_ coexistence.

## Introduction

Hydrogenation of CO_2_ into hydrocarbons has attracted intense interest as a route to produce value-added fuels^1,2^ and chemicals^3,4^ while mitigating CO_2_ emissions^5–9^. Modified iron-based Fischer–Tropsch synthesis (FTS) catalysts are particularly appealing for this reaction because of their activity, affordability, and inherent promotion of the water–gas shift reaction (WGS)^10–12^. Under typical CO_2_ hydrogenation conditions (300 – 360 °C, 1 – 5 MPa), iron catalysts tend to evolve into a mixture of iron carbide and iron oxide phases^13^. In fact, it is commonly observed in operando studies that iron initially carburizes to form carbides (e.g. χ-Fe_5_C_2_)^11,14^, but the H_2_O byproduct of CO_2_ conversion concurrently re-oxidizes some iron into magnetite (Fe_3_O_4_)^13^. Thus, the catalyst reaches a dynamic steady state comprising iron carbide and oxide phases, which synergistically couple FTS and reverse WGS. Consistent with this understanding, the vast majority of literature reports on Fe-based CO_2_ hydrogenation catalysts have identified Hägg iron carbide (χ-Fe_5_C_2_) as the predominant active phase, with magnetite (Fe_3_O_4_) playing a secondary but important role in maintaining activity^11,14^.

Emerging evidence underscores the catalytic importance of Fe_7_C_3_. Zhao et al. showed that all major iron carbides, including Fe_7_C_3_ are catalytically active in FTS^15,16^, and Chang et al. reported that Fe_7_C_3_ can exhibit the highest intrinsic turnover frequency among the iron carbides under typical FTS conditions^17^. These findings suggest that Fe_7_C_3_ could offer performance advantages over the traditional iron carbide phases, surpassing even χ-Fe_5_C_2_ in intrinsic activity, and thus merits special attention in catalyst design. In contrast, the role of the Fe_7_C_3_ phase in CO_2_ hydrogenation has remained largely unexplored^18^, primarily due to a historical research focus on χ-Fe_5_C_2_ and Fe_3_O_4_, as well as the practical challenge of obtaining Fe_7_C_3_ as a stable active phase under reaction conditions. Recent studies have begun to probe Fe_7_C_3_-based catalysts for CO_2_ hydrogenation, though with mixed^19^. Pasupulety et al. examined a K and Zn promoted iron catalyst supported on ZrO_2_ synthesized via a citric acid method with the aim of facilitating Fe_7_C_3_ formation. They reported that a CO/H_2_ pretreatment could indeed produce the Fe_7_C_3_ phase in this Fe–Zn–K/ZrO_2_ system; however, the activated catalyst was far from phase-pure, as a significant fraction of magnetite remained present alongside Fe_7_C_3_. The persistence of substantial Fe_3_O_4_ as well as the dominant ZrO_2_ support phase corresponded with suboptimal catalytic performance. The underwhelming results from such attempts highlight the need for new approaches to generate a purer and more stable Fe_7_C_3_ phase under CO_2_ hydrogenation conditions.

The scarcity of stable Fe_7_C_3_ in working catalysts arises from several intertwined factors. First, the thermodynamic and kinetic constraints governing phase formation often favor more common iron carbides or metallic iron under typical reaction temperatures and CO/H_2_ ratios^20,21^. Second, the complexity of Fe_7_C_3_’s formation from precursor materials often necessitates stringent pretreatment conditions that are not easily translated to large-scale or continuous operations^17^. Third, even when Fe_7_C_3_ is formed, maintaining its phase purity and stability under highly dynamic reaction environments, such as high partial pressures of water and oxidizing conditions inherent in CO_2_ hydrogenation, poses additional hurdles^13,22^.

In light of the gaps in prior research, we focus on Fe_7_C_3_ as a target active phase for CO_2_ hydrogenation and seeks to overcome the aforementioned stabilization challenges^21^. In contrast to previous works, we demonstrate that an in-situ carburization strategy can produce a nearly phase-pure Fe_7_C_3_ catalyst that remains stable under reaction conditions for prolonged periods. Notably, the Fe_7_C_3_-rich phase achieved in our work exhibits enhanced catalytic activity, including higher CO_2_ conversion and C_2+_^=^ olefin productivity compared to conventional Fe_3_O_4_/χ-Fe_5_C_2_-based catalysts. These findings represent the first clear evidence that Fe_7_C_3_ can serve as a durable and highly active phase for CO_2_ hydrogenation to hydrocarbons. Accordingly, the motivation of this study is to fill the knowledge gap regarding Fe_7_C_3_ by exploring its formation and function during CO_2_ hydrogenation. In this work, we aim to highlight a new pathway for designing iron-based CO_2_ hydrogenation catalysts beyond the traditional Fe_3_O_4_/χ-Fe_5_C_2_ paradigm, thereby advancing the development of more efficient CO_2_-to-fuels technologies.

## Results

### Structural characterizations

**Fig. 1:**
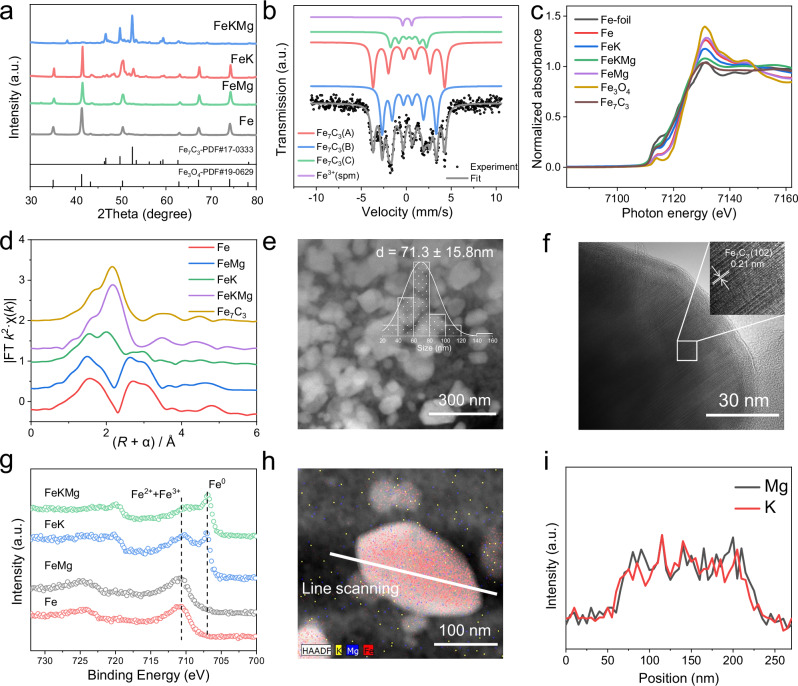
Comprehensive characterization of spent catalysts. XRD patterns of the spent Fe, FeMg, FeK, and FeKMg (**a**). Mössbauer spectrum of the spent FeKMg (**b**). Fe K-edge X-ray absorption near-edge structure (XANES) spectra (**c**). Fourier transform extended X-ray absorption fine structure (FT EXAFS) at the Fe K-edge (**d**) for the Fe, FeMg, FeK, and FeKMg. Particle size distribution (**e**) and HRTEM image (**f**) of the FeKMg catalyst. Fe 2*p* XPS profiles for the Fe, FeMg, FeK, and FeKMg catalysts (**g**). STEM-EDS elemental mapping (**h**) and line scanning analysis (**i**) of the FeKMg catalyst. All spent catalysts (Fe, FeMg, FeK, FeKMg) were collected after reaching steady-state catalytic performance under standard conditions (340 °C, 2 MPa, H_2_/CO_2_ = 3, GHSV = 6 L·g_cat_^−1^·h^−1^).

**Fig. 2:**
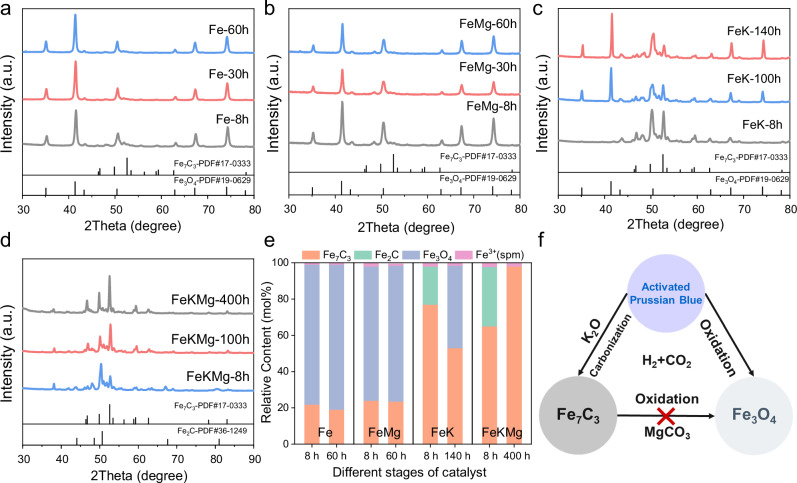
Phase evolution of catalyst during the reaction. **a** Fe, **b** FeMg, **c** FeK, **d** FeKMg. Reaction conditions: 0.10 g catalyst, 340 °C, GHSV = 6 L·g_cat_^−1^·h^−1^, 2 MPa, H_2_/CO_2_ = 3. Overview of the catalyst phase transitions observed during the reaction (**e**). Schematic illustration of promoters’ effect on catalyst phases (**f**).

Following our previously reported method for synthesizing Prussian blue analogue (PBA) precursors^23^, a series of PBA-based catalysts were synthesized and activated under an NH_3_ atmosphere. This NH_3_ atmosphere not only promotes the thermal decomposition of the PB analogues but also facilitates the formation of Fe_2_N as the primary phase in the fresh catalysts, as confirmed by X-ray diffraction (XRD) (Supplementary Fig. 1 and Fig. 2). Once subjected to CO_2_ hydrogenation, Fe_2_N rapidly undergoes an in situ transformation into iron oxide and iron carbide^24^, as illustrated in Fig. 1a. Specifically, the Fe and FeMg catalysts both display characteristic Fe_3_O_4_ peaks, indicating extensive oxidation of iron under the reaction conditions. Introducing potassium (FeK) induces partial carbide formation, and the subsequent addition of magnesium (FeKMg) leads to the clear emergence of a distinct Fe_7_C_3_ phase. Mössbauer spectroscopy and fitting analyses (Fig. 1b, Supplementary Fig. 3, Supplementary Table 1) corroborate the presence of Fe_7_C_3_ and reveal the composition of each spent catalyst^25^.

To gain deeper insights into the electronic structure, we employed X-ray absorption spectroscopy (XAS) and X-ray photoelectron spectroscopy (XPS). Fe K-edge XANES spectra (Fig. 1c) reveal that the spent Fe and FeMg catalysts have a pre-edge feature indicative of iron oxides. Introducing K shifts the absorption edge between oxidized and metallic states, while subsequent Mg addition drives a more pronounced shift toward lower energy, closely matching that of Fe_7_C_3_. This suggests that, under reaction conditions, Fe predominantly exists as the iron carbide in FeKMg^11,26^. The XAFS fitting results show that Fe and FeMg catalysts have the similar short-range structure with a Fe-O scattering path at 3.5 Å, whereas the presence of FeKMg maintains a resembling coordination number structure closer to Fe_7_C_3_ (Fig. 1d, Supplementary Fig. 4, Supplementary Table 2). Consistent with these findings, XPS results (Fig. 1g) show that Fe and FeMg retain oxidized surfaces, whereas FeK exhibits partial metallic character and FeKMg is dominated by metallic Fe signals^27^. Catalyst compositions (Supplementary Table 3) indicate that all catalysts possess similar Fe contents.

High-resolution transmission electron microscopy (HRTEM) shows the average crystallite size of the FeKMg catalyst is about 71.2 nm (Fig. 1e) and a lattice spacing of ~2.1 Å corresponding to the (102) plane of Fe_7_C_3_ (Fig. 1f). STEM-EDS mapping and line (Fig. 1h–i) scans indicate uniform element distribution. For comparison, TEM images of the spent Fe catalyst (Supplementary Fig. 5) reveal a uniform distribution of Fe and O (average particle size ~47.06 nm). Similarly, STEM-EDS mapping of FeK (Supplementary Fig. 6) and FeMg (Supplementary Fig. 7) show homogeneous elemental distributions. However, FeMg remains mostly Fe_3_O_4_, whereas FeK is a mixture of Fe_7_C_3_ and Fe_3_O_4_.

Figure 2 illustrates the structural evolution of the catalysts during CO_2_ hydrogenation. The initial Fe_2_N phase in Fe and FeMg catalysts (Fig. 2a and b) rapidly oxidizes to iron oxides within 8 hours. In contrast, FeK (Fig. 2c) initially undergoes carbonization to form iron carbides, but subsequently experiences partial re-oxidation, resulting in a mixed oxide–carbide phase. Notably, only FeKMg (Fig. 2d) maintains a stable carbide structure throughout the reaction, predominantly featuring the rarely reported Fe_7_C_3_ phase. Mössbauer quantification (Fig. 2e, Supplementary Fig. 8, Supplementary Table 4) confirms that potassium significantly promotes the carbonization process, while magnesium plays a key role in stabilizing the Fe_7_C_3_ phase. Figure 2f schematically summarizes the distinct roles of K and Mg during phase evolution. In our system, a small amount of Fe_7_C_3_ pre-exists in the fresh catalyst and acts as a nucleation base for further carburization. The metastable Fe_2_N gradually converts into Fe_7_C_3_ under the assistance of potassium. Meanwhile, magnesium plays a crucial role in maintaining the structural and chemical stability of the catalyst during CO_2_ hydrogenation. The thermodynamic phase diagram for iron carbide formation was simulated through theoretical calculations. Under low carbon chemical potential, the formation energies of different iron carbides are very close, suggesting a flat potential energy surface that facilitates facile phase transformations among them. The presence of Fe_7_C_3_ under these conditions indicates its thermodynamic viability and the possibility of stabilization even in carbon-lean environments (Supplementary Fig. 9).

Previous catalyst systems predominantly rely on the coexistence and dynamic equilibrium between χ-Fe_5_C_2_ and Fe_3_O_4_ phases, identifying χ-Fe_5_C_2_ as the primary active component^9^. In clear contrast, our FeKMg catalyst achieves nearly phase-pure Fe_7_C_3_ stabilization under realistic CO_2_ hydrogenation conditions. Comprehensive characterization methods explicitly confirm Fe_7_C_3_ as the dominant and stable catalytic phase, providing deeper mechanistic insights into the active site characteristics of iron carbide catalysts. Additionally, surface-sensitive XPS analysis detects minor dispersed Fe^3+^ oxide species (Fig. 1g) that remain undetectable in bulk analyses, suggesting these trace oxide species could beneficially contribute RWGS activity by forming highly dispersed and advantageous Fe_x_O sites on Fe_7_C_3_ surface.

### Catalytic performance

**Fig. 3:**
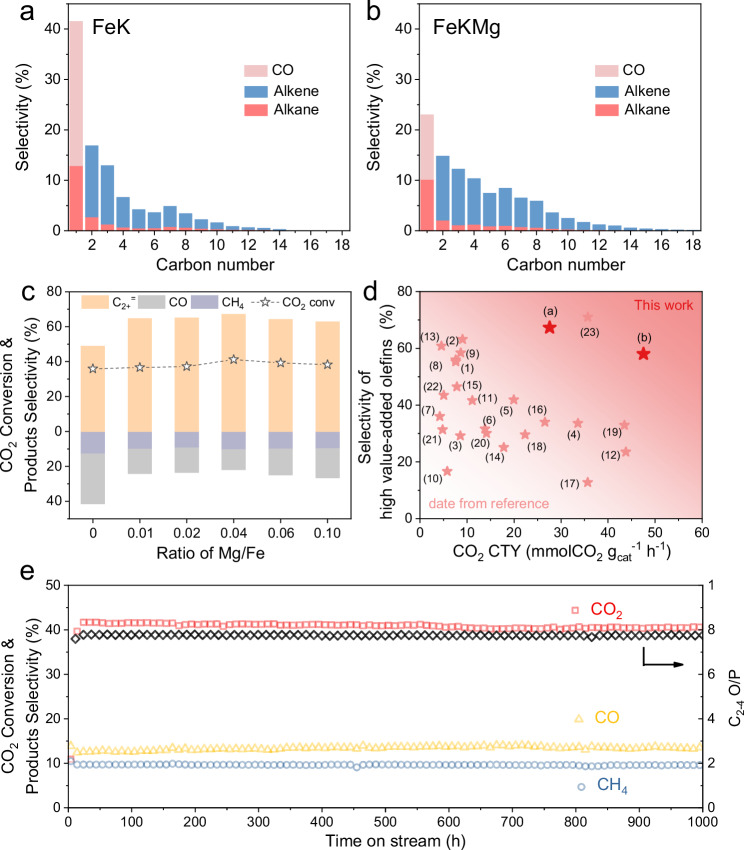
Catalytic performance. Product distributions from CO_2_ hydrogenation over Fe-based catalysts: **a** FeK, and **b** FeKMg. Bar charts show hydrocarbon selectivity versus carbon number. **c** CO_2_ conversion and product selectivity for Fe_7_C_3_-K with varying Mg levels. **d** Comparison of the catalytic performance of Fe_7_C_3_ with that of other previously reported catalysts (These catalysts cited from 1 to 23 are shown in supplementary Tables 8, high-value olefins: olefins of all carbon numbers). **e** Stability test of the Fe_7_C_3_-KMg catalyst. (Reaction conditions: 0.10 g of catalyst, 340 °C, 2.0 MPa, H_2_/CO_2_ = 3, GHSV = 6 L·g_cat_^−1^·h^−1^).

Beyond their structural differences, these catalysts exhibited notable disparities in catalytic behavior. To clearly demonstrate these differences, we compared their catalytic performance over the first 24 hours of time on stream (TOS) under identical reaction conditions. Table 1 summarizes the corresponding results, while Fig. 3a,b and Supplementary Fig. 10 showed the associated product distributions. The Fe and FeMg catalysts displayed similar behavior, each yielding a high fraction ( ~ 50%) of low-value products (CH_4_ and CO). Upon the introduction of potassium (FeK), olefin selectivity improved markedly, with high-value olefins reaching approximately 49.2%. Notably, despite initially high CO_2_ conversion across all four catalysts (Supplementary Fig. 11), catalysts without Mg gradually deactivated, concomitant with increased CO formation. This observation, together with structural analyses, indicates that the depletion of iron carbides, particularly Fe_7_C_3_, is a critical factor behind the decreasing FTS activity in later stages. In contrast, FeKMg consistently delivered higher CO_2_ conversion and lower selectivity to C_1_ by-products, ultimately achieving around 67.1% selectivity to high-value olefins.

**Table 1:** Catalytic performance for CO_2_ conversion over catalysts

Catalysts	Phase Composition [wt%]	CO_2_ Conversion [%]	C_1_ sel.[mol%]		C_2-4_ sel. [mol%]	C_5_^+^ sel. [mol%]	C_2-4_ O/P	C_2+_^=^ sel. [mol%]
			CO	CH_4_				
Fe	80%Fe_3_O_4_-19%Fe_7_C_3_	33.7	9.1	50.6	33.6	6.7	1.4	23.9
FeMg	74%Fe_3_O_4_-24%Fe_7_C_3_	34.0	8.0	47.5	36.2	8.3	1.2	25.8
FeK	46%Fe_3_O_4_-53%Fe_7_C_3_	35.9	28.6	12.9	36.4	22.1	6.6	49.2
FeKMg	98%Fe_7_C_3_	41.5	12.4	10.5	37.4	39.6	7.9	67.1

Reaction conditions: 340 °C, 2 MPa, 0.1 g catalyst, GHSV = 6 L·g_cat_^−1^·h^−1^, H_2_/CO_2_ = 3.

We next systematically explored the influence of reaction conditions and Mg loading on catalytic performance. Varying the Mg/Fe ratio led to an initial increase in CO_2_ conversion, followed by a decrease (Fig. 3c, Supplementary Table 5). The optimum Mg/Fe ratio of 0.04 yielded both the highest conversion and relatively low C_1_ by-product selectivity. XRD analyses (Supplementary Figs. 12 and 13, Supplementary Table 6) showed no significant change in catalyst phase with different Mg contents. Fe_7_C_3_ persisted as the main active phase, although MgCO_3_ appeared at higher Mg loadings. An elevated reaction temperature enhanced CO_2_ conversion but also boosted C_1_ by-product formation, while increasing the space velocity diminished conversion and concurrently raised the C_1_ fraction (Supplementary Fig. 14, Supplementary Tables 7 and 8). The Fe_7_C_3_-KMg catalyst exhibited stable performance across varying H_2_/CO_2_ ratios, and post-reaction XRD confirmed that Fe_7_C_3_ remained the dominant phase without notable Fe_3_O_4_ formation (Supplementary Fig. 15). Compared with previously reported catalysts (Fig. 3d, Supplementary Table 9), our Fe_7_C_3_-KMg catalyst demonstrates significantly higher olefin selectivity and minimal production of low-value by-products such as CH_4_ and CO. Under extended operation (340 °C, 2.0 MPa, H_2_/CO_2_ = 3, GHSV = 6 L·g_cat_^−1^·h^−1^), Fe_7_C_3_-KMg maintained exceptional stability over 1000 hours (Fig. 3e), exhibiting negligible changes in conversion and product distribution. Specifically, relative to recently reported alkali and alkaline-earth metal promoted iron catalysts^12,19^, our catalyst achieves comparable CO_2_ conversion ( ~ 41.5%) at notably milder conditions (2 MPa vs. 3 MPa) with superior olefin selectivity (67.1%) and enhanced long-term stability. This outstanding performance primarily results from the high phase purity of Fe_7_C_3_, distinctly superior to commonly reported χ-Fe_5_C_2_/Fe_3_O_4_ mixtures^9^, and from the synergistic promoting effects of potassium and magnesium. These characteristics underscore its robust potential for practical CO_2_ hydrogenation applications.

In summary, our results provide clear evidence that nearly phase-pure Fe_7_C_3_ catalysts derived from PBAs can efficiently drive CO_2_ hydrogenation to olefins with outstanding selectivity and long-term stability. These performance differences among catalysts underscore the critical role of phase purity and promoter interactions. The underlying structural mechanisms driving these catalytic outcomes, especially the stabilization effect of Mg and promotion effect of K, will be discussed in depth in subsequent sections.

### Structural information and mechanism of the magnesium promoter

To elucidate the structure-performance relationship in magnesium-promoted Fe_7_C_3_ catalysts, a systematic investigation of the magnesium species’ structure and spatial distribution within the catalytic system is critically needed. XPS quantification reveals that magnesium is predominantly localized on the catalyst surface, exhibiting a notably higher Mg/Fe atomic ratio compared to the bulk (Supplementary Fig. 16). Further XPS analyses confirm that Mg and K promoters primarily exist as surface MgCO_3_ and K_2_O species, respectively (Supplementary Fig. 17–18). Specifically, the characteristic K 2*p* peak around 292.7 eV clearly indicates the presence of surface-bound potassium oxide (K_2_O) rather than metallic potassium or lattice-incorporated potassium species, aligning well with literature consensus on potassium-promoted iron carbide catalysts^28,29^. Additionally, combined XPS analyses and DFT calculations indicate the absence of significant electronic interactions between the potassium and magnesium promoters (Supplementary Fig. 19).

**Fig. 4:**
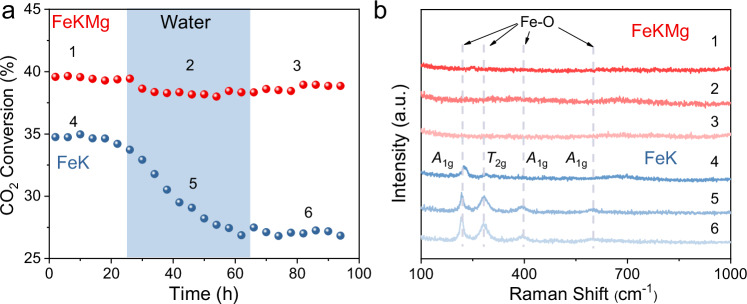
Effect of water on catalytic performance and surface structure. **a** CO_2_ hydrogenation activity under dry conditions and with an additional 10% gas-phase water (Reaction conditions: 0.10 g catalyst, 340 °C, 2.0 MPa, H_2_/CO_2_ = 3, GHSV = 6 L·g_cat_^−1^·h^−1^). **b** Corresponding Raman spectra recorded after each reaction stage (spectra 1–6 represent sequential stages of reaction).

Considering the critical influence of water formed during CO_2_ hydrogenation on catalyst stability, we specifically examined the impact of water exposure on catalyst performance (Fig. 4a and Supplementary Fig. 20). Whereas introducing H_2_O significantly decreased the activity of FeK, the FeKMg catalyst exhibited only minor performance fluctuations, indicating that Mg incorporation critically enhances catalyst resistance to water-induced deactivation. Even under high water partial pressures, FeKMg exhibited exceptional structural and catalytic stability. Raman spectra (Fig. 4b) corroborate this finding: although the FeK catalyst progressively lost surface carbon species and displayed emerging Fe–O vibrational peaks, FeKMg showed no notable oxidation-related signals.

To further elucidate the interactions between H_2_O and the catalyst, we performed D_2_O-TPD experiments (Supplementary Fig. 21) and transient kinetic analyses (Supplementary Fig. 22). TPD measurements revealed that both FeK and FeKMg catalysts exhibit similar D_2_O adsorption capacities; however, FeK produced a noticeably higher signal for dissociated D_2_. This suggests that the addition of Mg inhibit the dissociation of D_2_O. Moreover, we carried out transient kinetic experiments to quantify the catalyst’s capacity for D_2_O dissociation. By monitoring the D_2_ signal generated from the reaction between CO and surface-dissociated D_2_O, we were able to determine the amount of dissociated D_2_O on the catalyst surface. The FeKMg catalyst showed a lower quantity of dissociated D_2_O, further confirming that Mg suppresses D_2_O dissociation. This reduction in water splitting is a key factor in enhancing the stability of the FeKMg catalyst under reaction conditions, as it protects the active iron carbide phase from oxidation.

Both FeK and FeKMg catalysts exhibited remarkably high olefin selectivity. Previous studies have demonstrated that alkali metal promoters, particularly potassium, effectively enhance olefin selectivity by suppressing secondary olefin hydrogenation^30,31^. Our pulse experiment using propylene hydrogenation (Supplementary Fig. 23) explicitly confirmed this effect, clearly showing that potassium significantly inhibits olefin hydrogenation. In contrast, magnesium had minimal influence on this reaction. Thus, our findings indicate that the superior olefin selectivity observed is primarily attributed to the potassium promoter’s ability to effectively limit secondary hydrogenation of olefins.

**Fig. 5:**
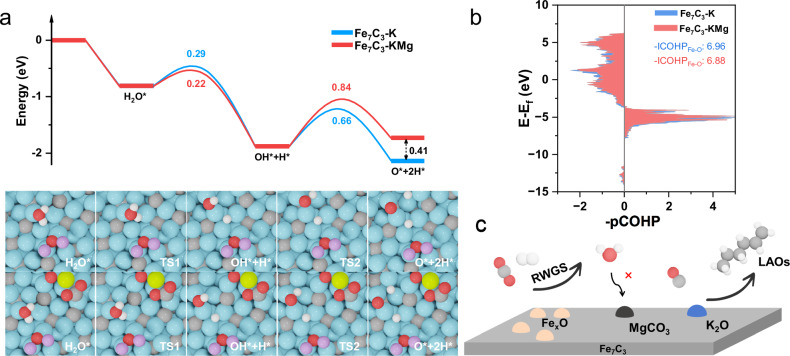
Mechanism of the Mg promoter. **a** Theoretical calculations in dissociation process of H_2_O on Fe_7_C_3_-K and Fe_7_C_3_-KMg. **b** Crystal orbital Hamilton population (COHP) analysis of Fe-O bonds on Fe_7_C_3_-K and Fe_7_C_3_-KMg. **c** Schematic illustration of the olefins synthesis reaction process via the Fe_7_C_3_-KMg catalyst system from CO_2_.

To clarify the atomic-scale mechanism underlying magnesium’s stabilizing effect, we performed DFT calculations to investigate water dissociation behavior on various catalyst surfaces. The results for Fe_7_C_3_ and Fe_7_C_3_-Mg are presented in Supplementary Fig. 24, while Fig. 5a shows the calculations for Fe_7_C_3_-K and Fe_7_C_3_-KMg. Adsorption energies of water on all four models are comparable, averaging approximately -0.80 eV, indicating similar water-binding strengths. Calculated energy barriers for the first O–H bond dissociation were 0.29 eV on Fe_7_C_3_-K and 0.22 eV on Fe_7_C_3_-KMg, indicating that initial dissociation occurs readily under reaction conditions. However, the subsequent dissociation of the second O–H bond is significantly more challenging, especially on Fe_7_C_3_-KMg, with an energy barrier increasing to 0.84 eV compared to 0.66 eV on Fe_7_C_3_-K. These calculations clearly illustrate magnesium’s substantial inhibitory effect on O–H bond cleavage.

Moreover, introducing magnesium significantly hampers oxygen adsorption, as evidenced by calculated reaction energies for water dissociation: −2.14 eV on Fe_7_C_3_-K and −1.73 eV on Fe_7_C_3_-KMg. Crystal orbital Hamilton population (COHP) analysis, which quantitatively measures bond strength through integrated COHP (-ICOHP) values (higher values indicate stronger bonding), further corroborates this observation. As depicted in Fig. 5b, the -ICOHP values for Fe–O bonds decrease from 6.96 on Fe_7_C_3_-K to 6.88 on Fe_7_C_3_-KMg, aligning with the reaction energy trends. Supplementary Fig. 25 shows analogous results for Fe_7_C_3_ and Fe_7_C_3_-Mg, with -ICOHP values of 7.03 and 6.95, respectively. These results collectively suggest that magnesium markedly reduces the oxygen adsorption capacity on Fe_7_C_3_ surfaces. Charge density difference analyses (Supplementary Fig. 26) further reveal electronic interactions: electrons transfer from K_2_O to Fe_7_C_3_ in Fe_7_C_3_-K, creating an electron-rich Fe_7_C_3_ surface favorable for oxygen adsorption and subsequent oxidation. Conversely, MgCO_3_ in Fe_7_C_3_-Mg acts as an electron acceptor, resulting in a charge-deficient Fe_7_C_3_ surface. Bader charge analysis quantifies this electron depletion, showing Fe_7_C_3_ loses approximately 1.03 electrons upon magnesium incorporation, thereby significantly mitigating oxygen adsorption and subsequent surface oxidation.

Integrating these findings, we propose the following catalytic mechanism (Fig. 5c): CO_2_ initially undergoes the RWGS reaction, facilitated by highly dispersed Fe_x_O species on the Fe_7_C_3_ catalyst surface, producing CO and water. Magnesium critically inhibits dissociation of the generated water, effectively preventing oxidation and subsequent deactivation of the active Fe_7_C_3_ phase. Consequently, in the presence of potassium promotion, the stable Fe_7_C_3_ surface efficiently catalyzes the conversion of CO and H_2_ into high-value olefins. This synergistic interplay between magnesium stabilization and potassium promotion underlies the outstanding selectivity and long-term stability of the Fe_7_C_3_-KMg catalyst. Nevertheless, despite the intrinsic oxidation resistance provided by Mg, accumulation of water under prolonged industrial operation could still pose challenges to catalyst stability. Therefore, complementary reactor-level engineering strategies may be required for industrial applications. Specifically, strategies such as membrane-assisted in-situ water removal, downstream condensation coupled with recycle loops, and structured or hydrophobic reactor designs to reduce water retention have been successfully applied in catalytic processes. Integrating these reactor-level approaches with the intrinsic material-level oxidation resistance provided by Mg could yield synergistic improvements, significantly enhancing the practical viability of Fe_7_C_3_-based catalysts under industrially relevant conditions.

## Discussion

In summary, our study introduces a novel synthesis strategy for the Fe_7_C_3_ phase that leverages the unique roles of K and Mg promoters. By extracting Fe_7_C_3_ seeds from Prussian blue and employing K to enhance carbonization while using Mg to mitigate water-induced oxidation, we have successfully stabilized the Fe_7_C_3_ phase under demanding CO_2_ hydrogenation conditions. Our Fe_7_C_3_-KMg catalyst exhibits catalytic performance that far exceeds that of conventional iron catalysts and opens up new avenues for the innovative design of CO_2_ hydrogenation catalysts. Moreover, we have not only developed an efficient method to synthesize single-phase Fe_7_C_3_ but also elucidated the intrinsic link between the structure of Fe_7_C_3_ and its catalytic performance, thereby providing a solid foundation for further catalyst optimization. Future work will focus on optimizing this strategy further and exploring its applicability across a broader range of reaction conditions, paving the way for more sustainable and efficient CO_2_ utilization technologies.

## Methods

### Catalyst preparation

All catalysts containing different promoters were synthesized by a coprecipitation method, using Fe(NO_3_)_3_·9H_2_O as the iron source, and either (NH_4_)_4_Fe(CN)_6_ or K_4_[Fe(CN)_6_] as the precipitating agent. A suitable amount of polyvinylpyrrolidone K30 (average molecular weight ~40,000; TCI, Shanghai) was dissolved in 200 g of deionized water to ensure uniform dispersion and controlled nucleation of the PBA precursors. Subsequently, a solution of either (NH_4_)_4_Fe(CN)_6_ (Honeywell Specialty Chemicals Seelze GmbH) or K_4_[Fe(CN)_6_] (solution A) was prepared. Separately, Fe(NO_3_)_3_·9H_2_O (AR, Sinopharm Chemical Reagent Co., Ltd.) was dissolved in 200 g of deionized water (solution B). Solution B was then slowly added into solution A under vigorous stirring to form the catalyst precursor. Finally, different amounts of Mg(NO_3_)_2_·4H_2_O were introduced via incipient wetness impregnation. The final Fe/Mg ratio of each sample was determined by inductively coupled plasma-optical emission spectrometry (ICP-OES, Perkin Elmer). Catalyst samples are named based on the promoters employed: Fe refers to the iron catalyst without any promoter; FeMg refers to the catalyst modified with magnesium only; FeK denotes the catalyst promoted solely with potassium; and FeKMg indicates the catalyst co-promoted with both potassium and magnesium.

Specifically, the general designation “FeKMg” is used throughout the manuscript to describe the co-promoted catalyst. The notation “Fe_7_C_3_-KMg” is explicitly employed only when the presence of the Fe_7_C_3_ carbide phase has been confirmed through structural characterization.

### Catalyst characterizations

XRD patterns were acquired using a Bruker D8 powder diffractometer located in Karlsruhe, Germany, with Co Kα radiation (λ = 0.179 nm). The instrument operated at a voltage of 35 kV and a current of 40 mA. A continuous scan mode was employed with a step size of 0.04° and a dwell time of 0.4 seconds, covering a 2θ range from 40° to 75°.

TEM analysis was performed using an FEI Talos F200A electron microscope operating at 200 kV. The samples were sonicated in ethanol, then deposited onto a copper grid with a porous carbon film. Before testing, the samples were irradiated with an infrared lamp for 15 minutes to remove any residual solvents.

HAADF-STEM was used to capture STEM images (2048 × 2048 pixels) with a camera length of 260 mm and a spot diameter of 0.5 nm.

XPS spectra were recorded using a Thermo Scientific K-alpha system with Al Kα radiation (hν = 1486.6 eV) as the X-ray source. To prevent oxidation, the samples were prepared in a glove box. The C 1 *s* peak (284.6 eV) was used as a reference for calibration.

PTH experiments for C_3_H_6_ were conducted using an AMI-300 apparatus equipped with a mass spectrometer. The catalysts were activated under ammonia gas, then switched to a 10% H_2_/Ar flow (50 mL/min), with the temperature set to 340 °C. C_3_H_6_ was pulsed into the system to complete the PTH. The effluent was monitored for C_3_H_6_ (m/z = 42) and C_3_H_8_ (m/z = 44) using a PFEIFFER Omnistar mass spectrometer.

XAFS data were collected at the BL14W1 beamline of the Shanghai Synchrotron Radiation Facility (SSRF), China, operating at 3.5 GeV with a maximum current of 260 mA. Energy calibration was performed using the absorption edge of pure Fe foil, and XAFS data were acquired in fluorescence mode.

Mössbauer experiments were conducted using an MR-351 constant acceleration transmission spectrometer at 10 K with 25 mCi 57Co in a Rh matrix. Phase composition was identified based on isomer shift (IS), quadrupole splitting (QS), and magnetic hyperfine field (H_hf_) parameters. The content of each phase was determined from the absorption peak areas, assuming the same recoil-free factor for all types of iron nuclei in the catalyst.

### Theoretical calculations

For calculation models, a periodical (1×1) of *h*-Fe_7_C_3_(211) slab was truncated from the optimized bulk phase, which contains 56 Fe and 23 C atoms. During the structural optimization, the bottom 27 Fe and 11 C atoms were fixed in the equilibrium positions as in the bulk phase while the others were allowed to relax. We selected the (211) facet for DFT modeling based on prior studies showing this to be a relatively low-index, catalytically active surface with moderate surface energy^32,33^. Additionally, the (211) plane exposes coordinatively unsaturated Fe atoms, suitable for CO_2_/H_2_ adsorption and promoter interaction modeling. A MgCO_3_ cluster was loaded on the *h*-Fe_7_C_3_(211) facet to represent the Mg-promoted catalyst. All spin-polarized calculations were carried out with VASP code^34,35^. The frozen-core projector-augmented wave (PAW)^36^ pseudo-potential with a cutoff energy of 450 eV was selected for the plane-wave expansion. The generalized gradient approximation in the Perdew-Burke-Ernzerhof (GGA-PBE)^37^ with van deer Waals correction (D3)^38^ was employed to describe the exchange-correlation energy. The convergence criteria for the force and electronic self-consistent iteration were set to 0.03 eV/Å and 10^−5^ eV, respectively. Gamma-centered (2×2×1) k-point was used for sampling of Brillouin zone. In all calculations, adsorption energies (*E*_ads_) were calculated based on *E*_ads_ = *E*_x/slab_ – [*E*_slab_ + *E*_x_], where *E*_x/slab_ is the total energy of the slab with adsorbents after full relaxation, *E*_slab_ is the total energy of the bare slab, and *E*_x_ is the total energy of the free adsorbents in the gas phase. Therefore, the more negative the *E*_ads_, the stronger the adsorption. Reaction energies (Δ*E*) were defined as Δ*E* = *E*_final_ - *E*_initial_, where *E*_final_ and *E*_initial_ represent the final state energy and initial state energy, respectively. Therefore, a negative Δ*E* represents an exothermic process. All transition states were calculated using the climbing image nudged elastic band method (CI-NEB)^39^, with the stretching frequencies analyzed in order to characterize whether a stationary point is a transition state with only one imaginary frequency.

### Catalyst testing

The catalytic efficiency of synthesized materials was systematically assessed using a quad-channel fixed-bed microreactor system. For each test run, 100 mg of catalyst was uniformly mixed with 200 mg quartz sand and packed into a quartz reaction chamber (10 mm ID) equipped with a temperature-monitoring stainless-steel sleeve. Standard evaluation parameters were maintained at 340 °C, 2.0 MPa, H_2_/CO_2_ = 3, and 6000 mL·g_cat_^−1^·h^−1^ unless otherwise specified. Post-reaction products underwent phase separation through sequential hot (160 °C) and cold (0 °C) trapping systems, enabling collection of solid waxes, liquid hydrocarbons, and aqueous phases for offline analysis via HP-PONA 19091s-001 chromatography.

Continuous gas monitoring was achieved through Agilent 7890B GC with specialized detection modules: Gaspro-FID assemblies resolved C1-C4 hydrocarbons, while a PONA-FID system characterized C4-C7 compounds. Gas composition of H_2_/CO_2_/CO/Ar was determined using coupled PLOT/Q, 5 Å molecular sieve, and Haysep Q columns interfaced with TCD detection. Carbon-based mass balance calculations confirmed experimental accuracy within ± 5% deviation through systematic comparison of inlet/outlet carbon fluxes.

The CO_2_ conversion (X_CO2_), product selectivity (S_i_), reaction rate (R) and C_2+_ olefin selectivity (${{{\rm{S}}}}_{\,{{{\rm{C}}}}_{2+}^{=}}$) were calculated by the following equations:

1 $${X}_{{{CO}}_{2}}=\frac{{{{CO}}_{2}}_{{in}}/{Ar}_{{in}}-{{{CO}}_{2}}_{{out}}/{{Ar}}_{{out}}}{{{{CO}}_{2}}_{{in}}/{{Ar}}_{{in}}}\times 100\%$$

2 $${S}_{i}=\frac{{N}_{i}\times {n}_{i}}{\sum \left({N}_{i}\times {n}_{i}\right)}\times 100\%$$

3 $${R}_{{{CO}}_{2}}=\frac{{GHSV}\times {X}_{{{CO}}_{2}}\times {C}_{{{CO}}_{2}}}{22400}\times 100\%$$

4 $${S}_{\,{C}_{2+}^{=}}=\frac{\sum {S}_{i}^{=}}{\sum \left({N}_{i}\times {n}_{i}\right)}\times 100\%$$

where CO_2 in/out_ denote molar flows of carbon dioxide at reactor feed and effluent streams respectively, Ar _in/out_ refer to molar flows of argon at the reactor inlet and outlet. S_i_ represents carbon-specific selectivity for product i, N_i_ indicates molar fraction, n_i_ corresponds to carbon count per molecule and S_i_^=^ is the specifically quantifies olefinic selectivity for i-carbon unsaturated hydrocarbons. GHSV corresponds to gas hourly space velocity. This analytical framework ensures comprehensive characterization of catalytic behavior while maintaining strict adherence to standardized evaluation protocols in heterogeneous catalysis research.

## Supplementary information

Supplementary Information

Transparent Peer Review file

## Source data

Source Data

## Data Availability

The data that support the findings of this study are available within the paper and its Supplementary Information, and all data are also available from the corresponding authors upon request. Source data are provided with this paper.
