# Supplementary material for: Stabilized Fe7C3 catalyst with K–Mg dual promotion for robust CO2 hydrogenation to high-value olefins
Source: Nat Commun. 2025 Aug 28;16:8044. doi: 10.1038/s41467-025-63218-3 (PMC12394697; doi:10.1038/s41467-025-63218-3)
Supplement: Supplementary file 1 — Supplementary Information [file 41467_2025_63218_MOESM1_ESM.pdf]

Supplementary Information

Stabilized Fe_7C_3 Catalyst with K–Mg Dual Promotion for Robust CO_2 Hydrogenation to High-Value Olefins

Fei Qian[1,2,3 †], Maolin Wang[4 †], Zidu Wei[2], Yi Cai[1,2,3], Zeping Sun[2], Ruikang Liang[2], Guangbo Liu[5], Ming Qing[2], Hong Wang[2], Jinjia Liu[1,2] *, Xing-Wu Liu[1,2] *, Yong Yang[1,2,3], Xiao-Dong Wen[1,2,3] *

[1]State Key Laboratory of Coal Conversion, Institute of Coal Chemistry, Chinese Academy of Sciences, Taiyuan, 030001, China; [2]National Energy Center for Coal to Liquids, Synfuels China Co., Ltd., Huairou District, Beijing, 101400, China; [3]University of Chinese Academy of Sciences, No. 19A Yuquan Road, Beijing, 100049, PR China; [4]Beijing National Laboratory for Molecular Sciences, New Cornerstone Science Laboratory, College of Chemistry and Molecular Engineering, Peking University, Beijing, China. [5]Key Laboratory of Photoelectric Conversion and Utilization of Solar Energy, Qingdao Institute of Bioenergy and Bioprocess Technology, Chinese Academy of Sciences, Qingdao 266101, China

[†]These authors contributed equally: Fei Qian, Maolin Wang

[*]Corresponding author: Email: liujinjia@synfuelschina.com.cn; liuxingwu@sxicc.ac.cn; wxd@sxicc.ac.cn

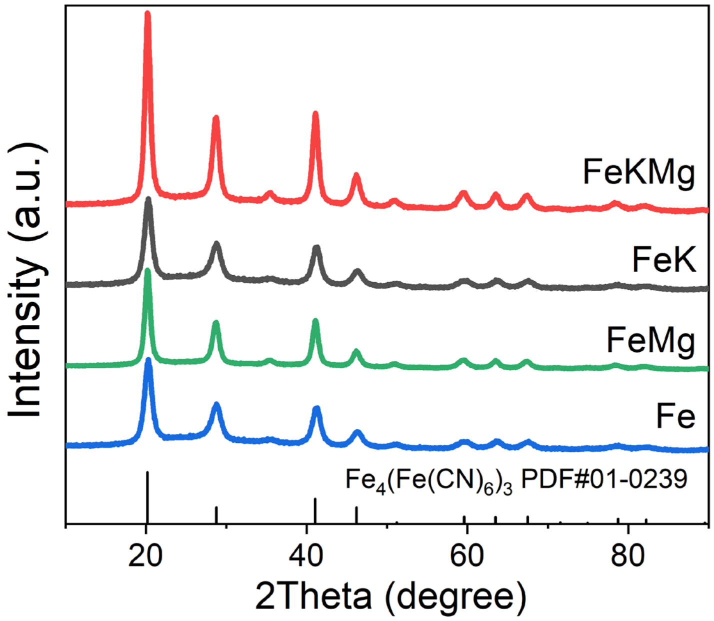

Supplementary Fig. 1 | XRD patterns of Fe-based catalysts and their modified variants. (Fe, FeMg, FeK, and FeKMg). The reference pattern of $Fe_4[Fe(CN)_6]_3$ (PDF#01-0239) is shown at the bottom for comparison.

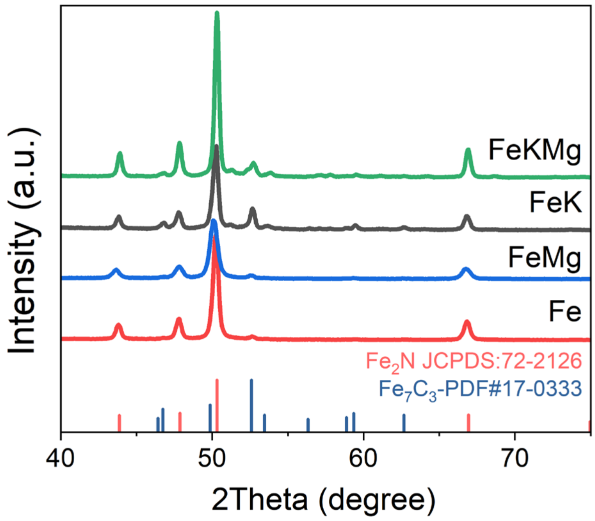

Supplementary Fig. 2 | XRD patterns of activated Fe-based catalysts. Fe, FeMg, FeK, and FeKMg. Activation conditions: 50 mL $NH_3 \cdot g_{cat}^{-1} \cdot min^{-1}$, 550 °C, 1 h.

Note: Regarding scalability and safety considerations of the NH_3 pretreatment at 550 °C used to form the Fe_2N precursor, this procedure was conducted under carefully controlled laboratory conditions without any abnormal pressure fluctuations or safety concerns observed. The NH_3 pretreatment gases were safely managed using standard gas handling and tail-gas absorption systems. Although NH_3 nitridation inherently involves higher temperatures compared to conventional CO/H_2 carburization methods, it provides precise control over precursor phase formation, significantly enhancing catalytic phase selectivity. The effectiveness and safety of NH_3 pretreatment strategies have been previously validated in literature[1, 2].

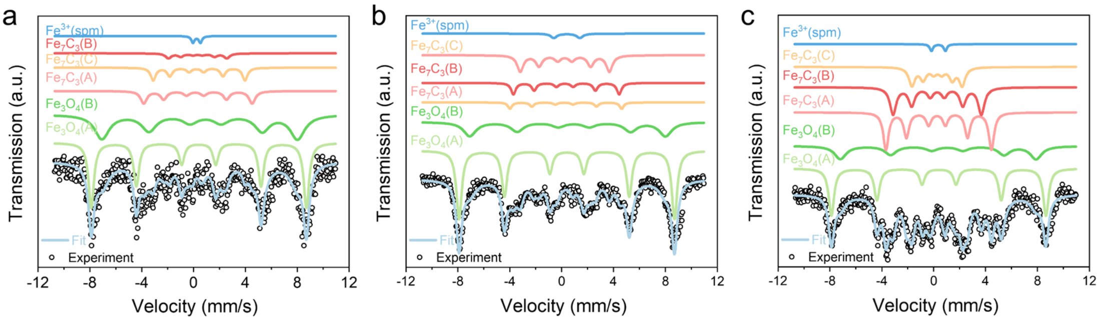

Supplementary Fig. 3 | Mössbauer spectra of the spent catalyst (a: Fe, b: FeMg, c: FeK). (Mössbauer experiments were conducted using an MR-351 constant acceleration transmission spectrometer at 10 K with 25 mCi 57Co in a Rh matrix.)

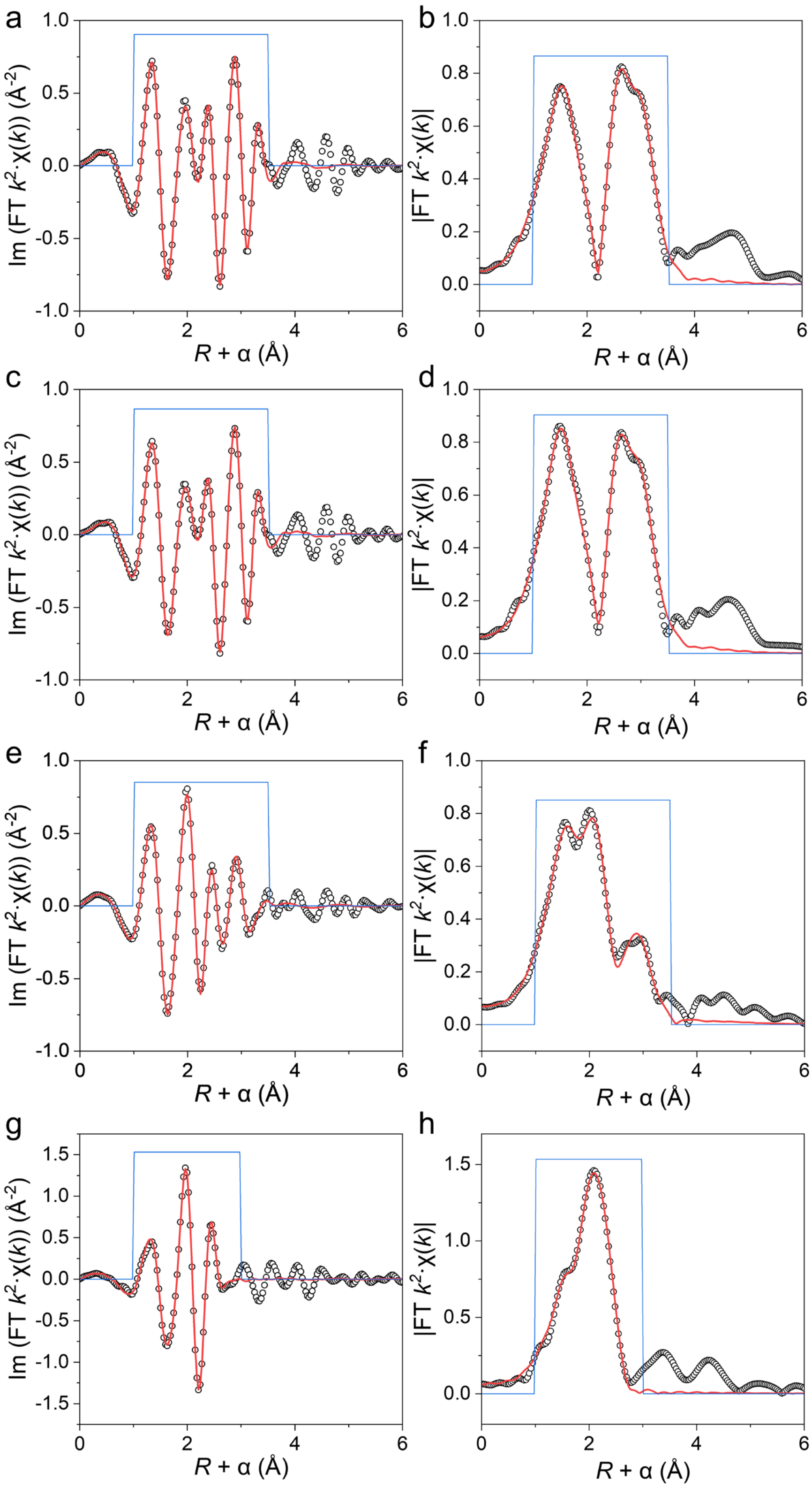

Supplementary Fig. 4 | Curve-fit (red line) of Fe *K*-edge EXAFS (black dot). Fe: (a) *R*-space imaginary part and (b) *R*-space; FeMg: (c) *R*-space imaginary part and (d) *R*-space. FeK: (e) *R*-space imaginary part and (f) *R*-space; FeKMg: (g) *R*-space imaginary part and (h) *R*-space. The blue line is the fitting windows. The data are k^2-weighted and without phase correction.

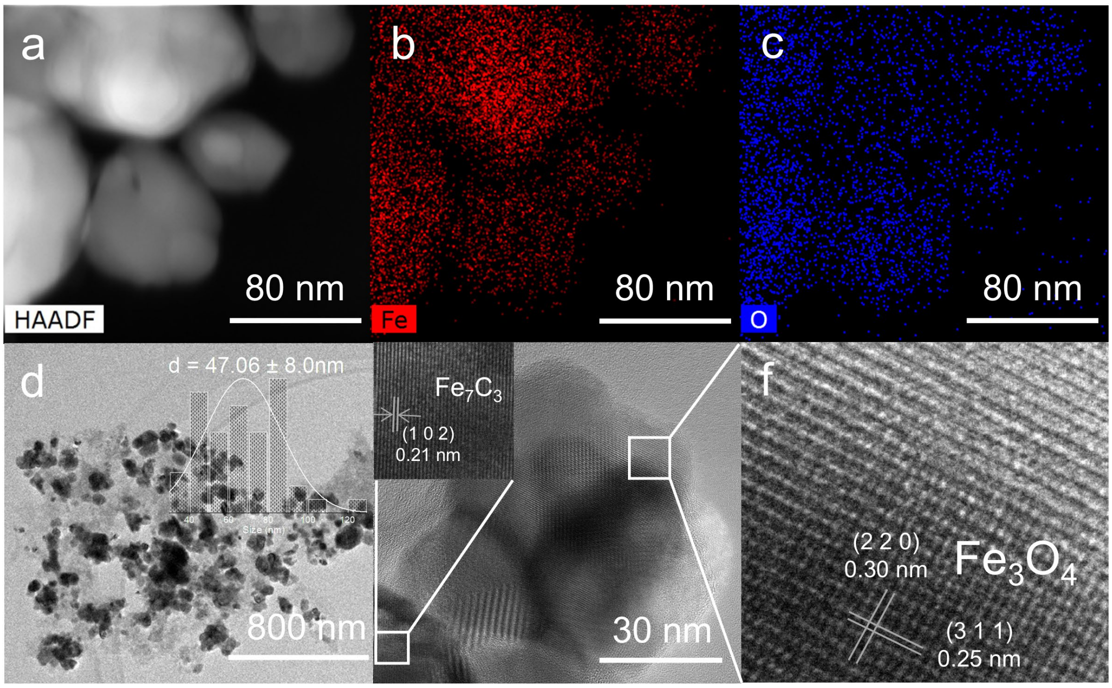

Supplementary Fig. 5 | HAADF (a), STEM-EDS elemental mapping images (b-c), particle size distribution (d) HRTEM image and FFT images of the HRTEM images (f) of Fe.

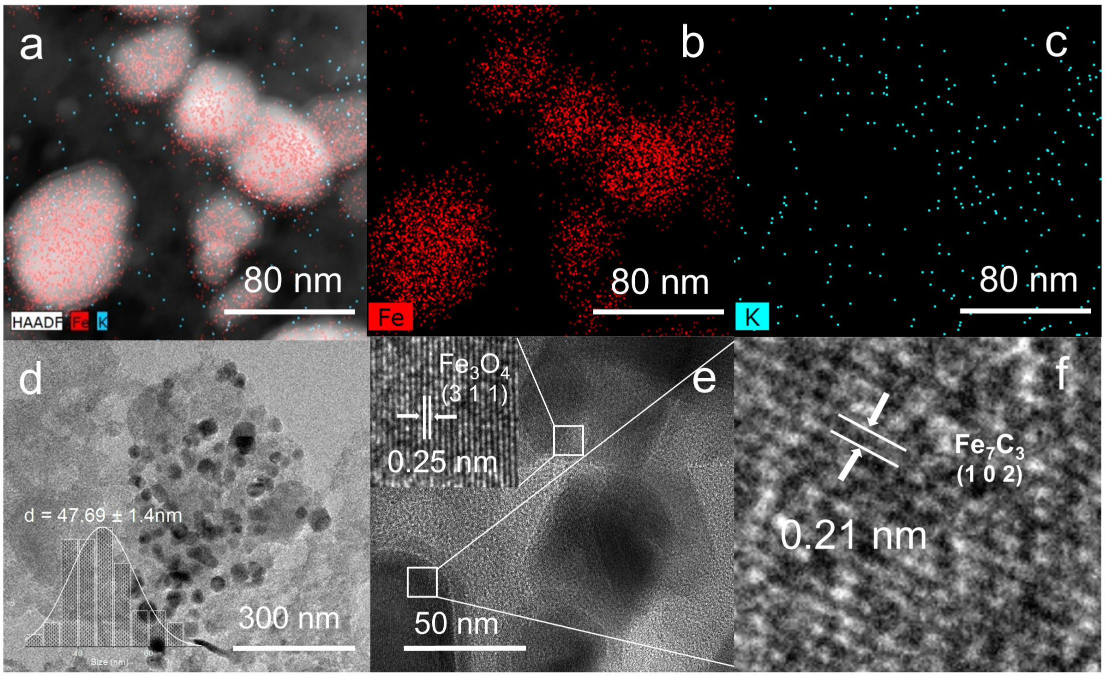

Supplementary Fig. 6 | HAADF (a), STEM-EDS elemental mapping images (b-c), particle size distribution (d) HRTEM image (e-f) of FeK.

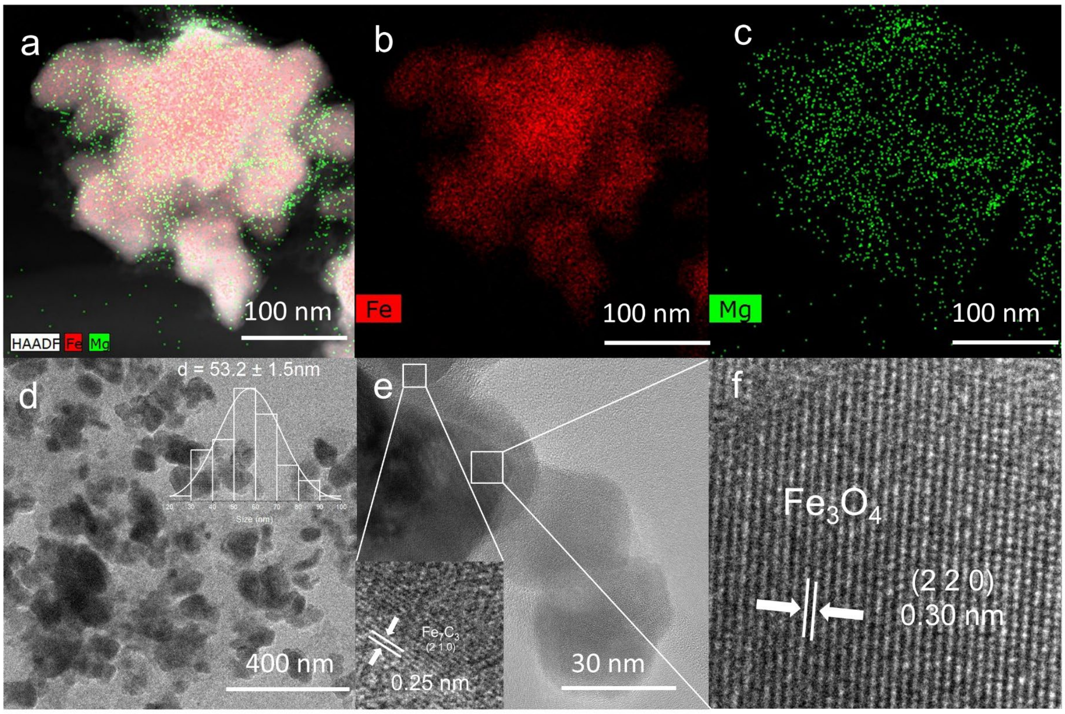

Supplementary Fig. 7 | HAADF (a), STEM-EDS elemental mapping images (b-d), particle size distribution (e) HRTEM image (f) of FeMg.

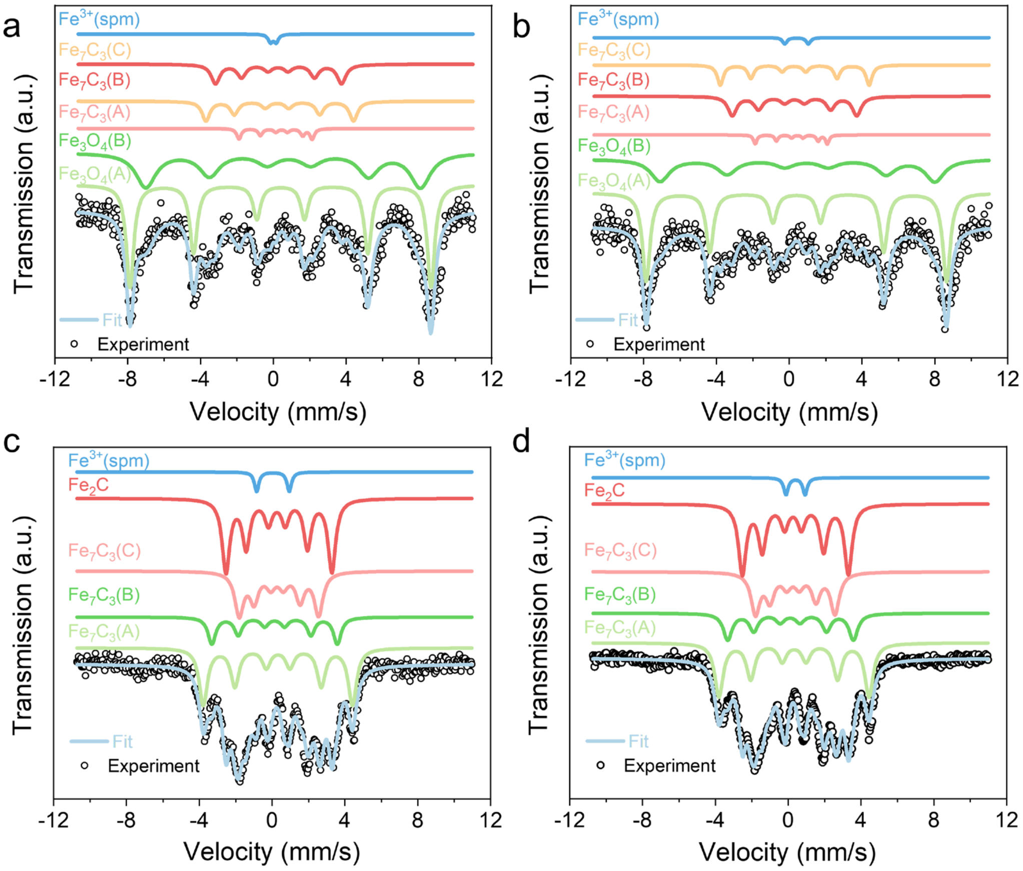

Supplementary Fig. 8 | Mössbauer spectra of the spent catalyst in 8 h. a: Fe, b: FeMg, c: FeK, d: FeKMg (Mössbauer experiments were conducted using an MR-351 constant acceleration transmission spectrometer at 10 K with 25 mCi 57Co in a Rh matrix.)

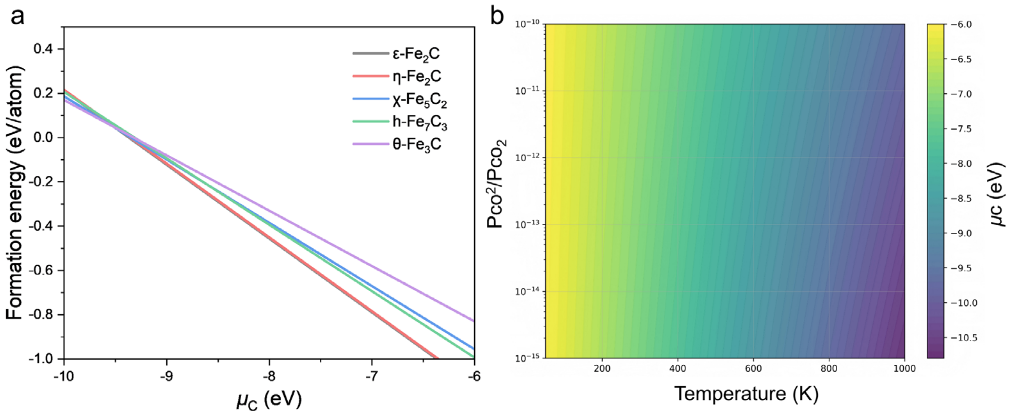

Supplementary Fig. 9. | (a) The calculated formation energy (eV/atom) of iron carbides with carbon chemical potential (μ_C). (b) The carbon chemical potential (μ_C) with temperature and partial pressure.

The calculations reveal that two Fe_2C phases exhibit the lowest formation energy, followed by Fe_7C_3, Fe_5C_2, and Fe_3C. Specifically, when $\mu_C < -8.0$ eV, Fe_7C_3 and Fe_5C_2 show comparable thermodynamic stability. Under low carbon chemical potential, the formation energies of different iron carbides are very close, suggesting a flat potential energy surface for iron carbides and thus facilitating facile phase transformations among them. Whereas for $\mu_C > -8.0$ eV, Fe_7C_3 demonstrates a lower formation energy than Fe_5C_2, indicating its superior thermodynamic stability. In Figure R2b, we plot the contour of carbon chemical potential as a function of temperature and pressure. Here, μ_C is defined based on the disproportionation reaction ($2CO = CO_2 + C$), such that $\mu_C = 2\mu_{CO} - \mu_{CO2}$. Higher temperatures and lower CO partial pressures result in lower carbon chemical potentials. It provides a direction for experiment to control synthesis desirable phases by modulating temperature and pressure. It is important to emphasize that while kinetic factors play a critical role in iron carbide phase transformations, they remain challenging to address in purely theoretical studies, representing a direction for future experimental-computational integration.

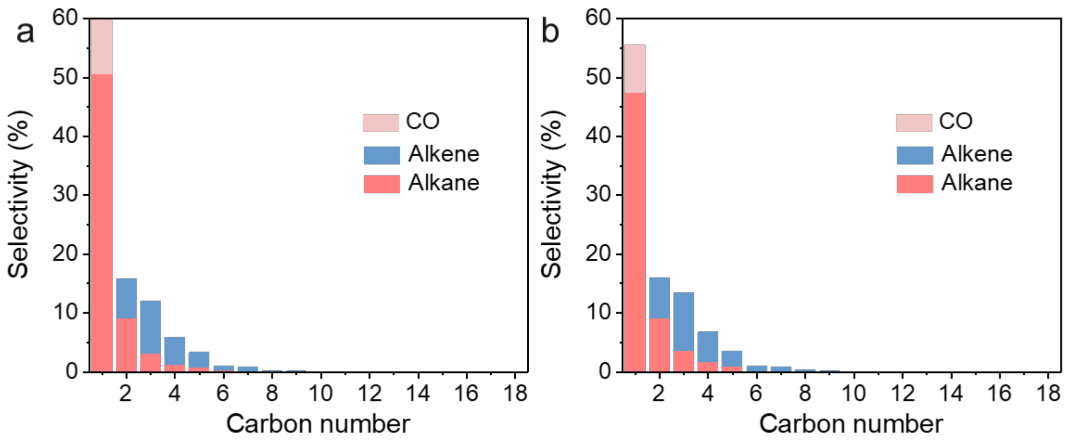

Supplementary Fig. 10 | Product distributions from CO_2 hydrogenation over Fe-based catalysts: (a) Fe, (b) FeMg, Bar charts show hydrocarbon selectivity versus carbon number, with pie charts indicating the fraction of high-value products (red) and others (grey). (Reaction conditions: 0.10 g of catalyst, 340 °C, 2.0 MPa, $H_2/CO_2 = 3$, GHSV = 6 $L \cdot g_{cat}^{-1} \cdot h^{-1}$)

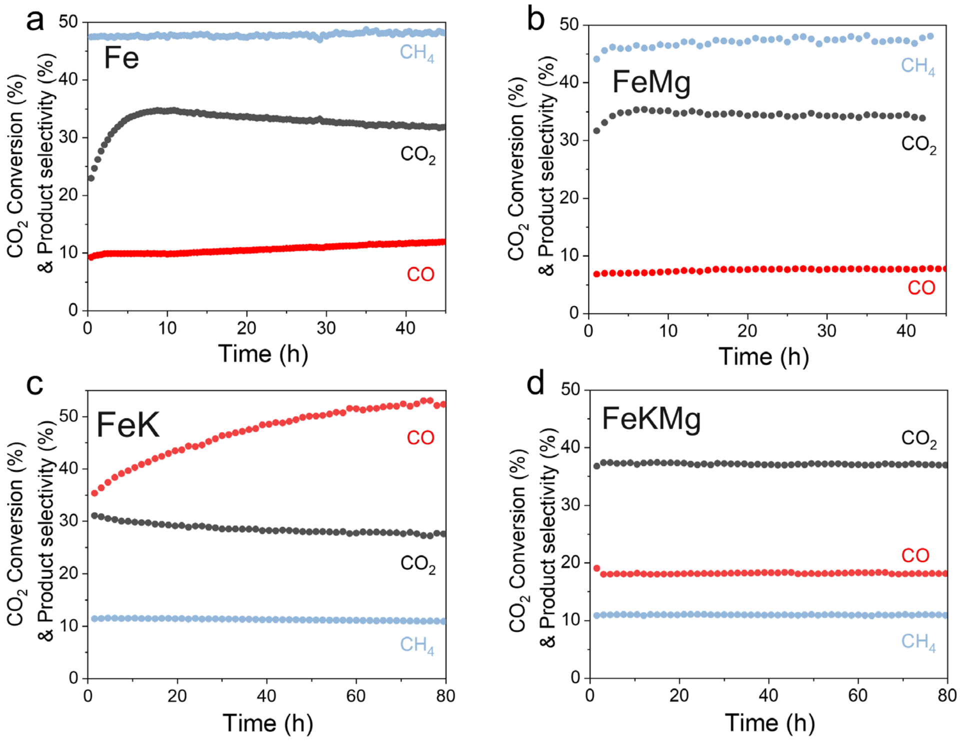

Supplementary Fig. 11 | Time-on-stream CO_2 conversion and product selectivity over different Fe-based catalysts: (a) Fe, (b) FeMg, (c) FeK, and (d) FeKMg. Data shows CO_2 conversion (black), CO selectivity (red), and CH_4 selectivity (blue) during stability tests. Reaction conditions: 0.10 g of catalyst, 340 °C, 2.0 MPa, $H_2/CO_2 = 3$, GHSV = 10 $L \cdot g_{cat}^{-1} \cdot h^{-1}$.

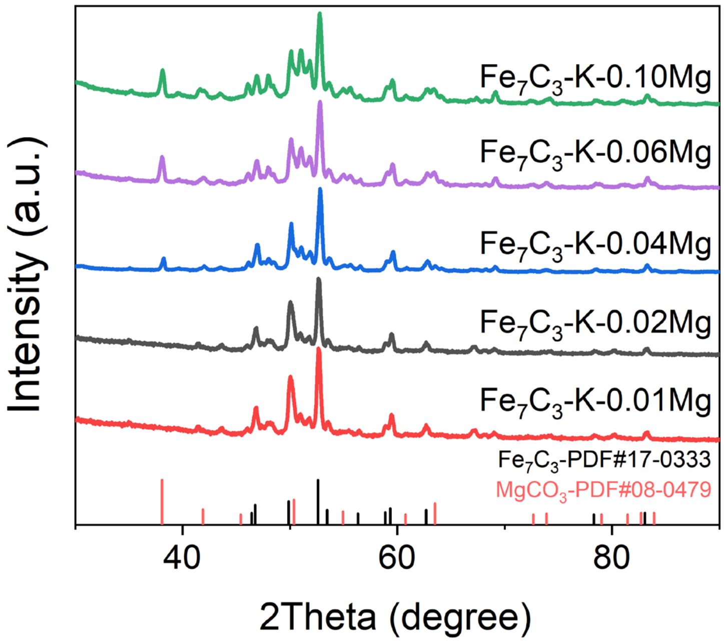

Supplementary Fig. 12 | XRD patterns of post-reaction Fe_7C_3 catalysts with varying Mg content: Fe_7C_3-K-0.01Mg, Fe_7C_3-K-0.02Mg, Fe_7C_3-K-0.04Mg, Fe_7C_3-K-0.06Mg, and Fe_7C_3-K-0.10Mg. The reference pattern of Fe_7C_3 (PDF#17-0333) is included for phase identification.

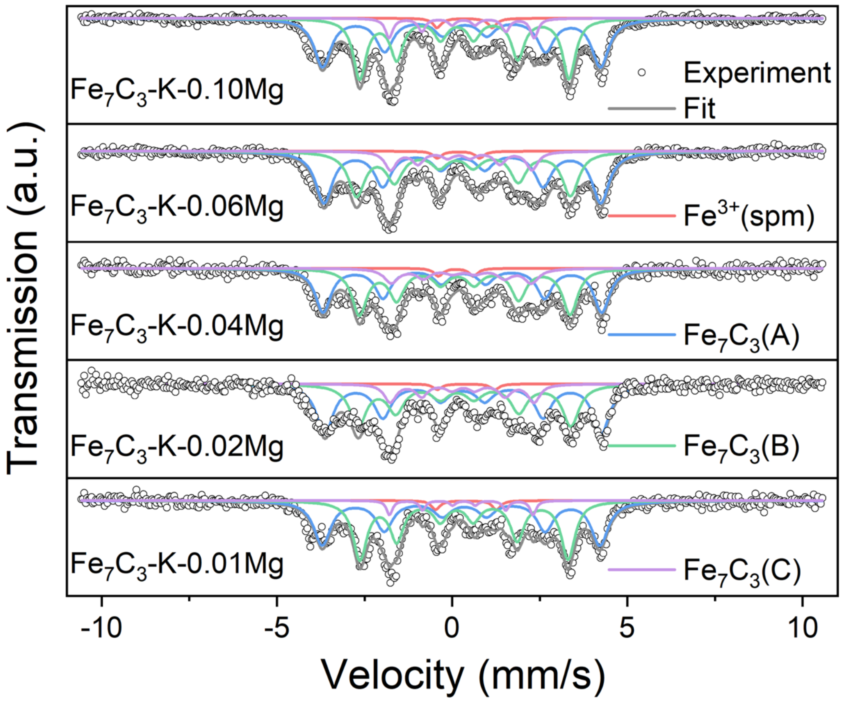

Supplementary Fig. 13 | 57Fe Mössbauer spectra and fitting results for post-FTS Fe_7C_3-K catalysts with varying Mg content (Fe_7C_3-K-0.01Mg to Fe_7C_3-K-0.10Mg). Experimental data (dots) were fitted (black lines) with multiple components: Fe^{3+} species (spm) and three different Fe_7C_3 carbide phases (A, B, and C).

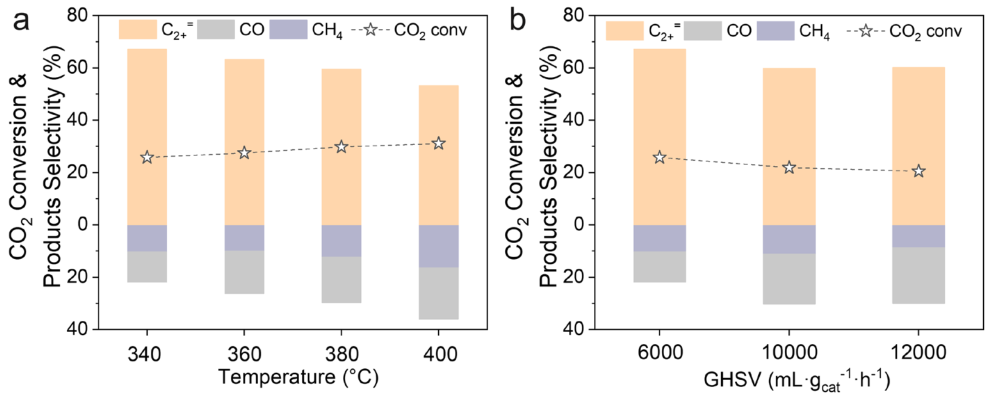

Supplementary Fig. 14 | Catalytic Performance. (a) Conversion of CO_2 and product selectivity of Fe_7C_3 with different temperatures. Reaction conditions: 0.10 g catalyst, GHSV= 6 $L\cdot g_{cat}^{-1}\cdot h^{-1}$, 2 MPa and H_2/CO_2 ratio of 3. (b) Conversion of CO_2 and product selectivity of Fe_7C_3 with different GHSV. Reaction conditions: 0.10 g catalyst, 340°C, 2 MPa and H_2/CO_2 ratio of 3.

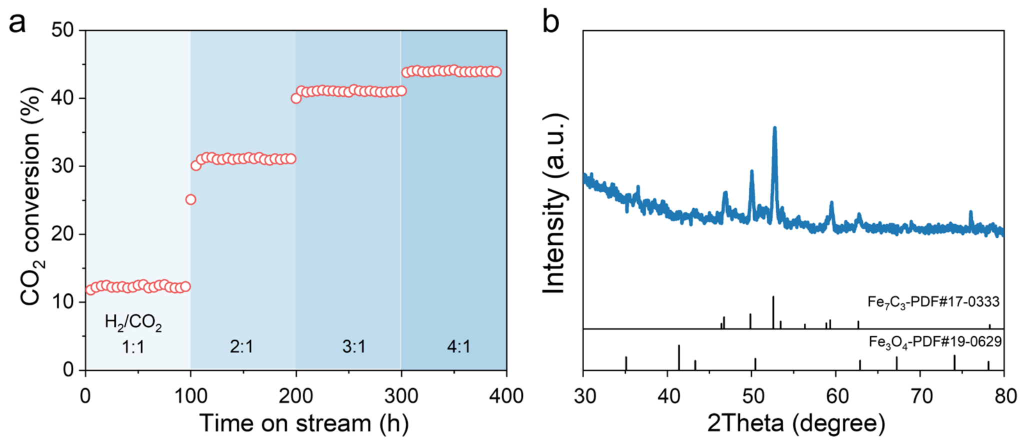

Supplementary Fig. 15 | (a) Conversion of the Fe_7C_3-KMg catalyst under fluctuating CO_2/H_2 ratios (1-4); (b) XRD patterns of the catalyst after reaction.

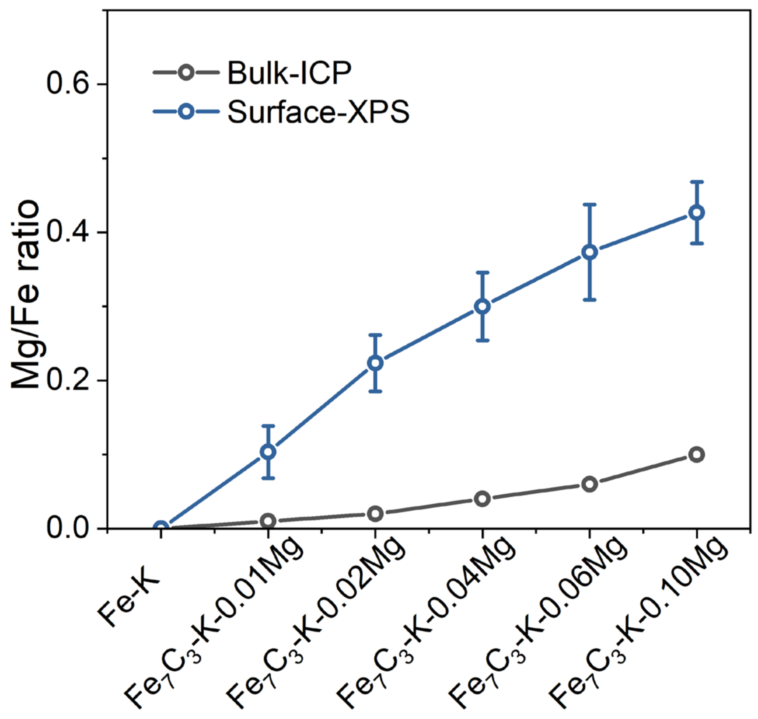

Supplementary Fig. 16 | Surface and bulk Mg/Fe ratios of catalysts for Fe_7C_3 with various addition magnesium levels. Error bars show standard deviation of peak intensities measured at n=3 random locations on the sample surface.

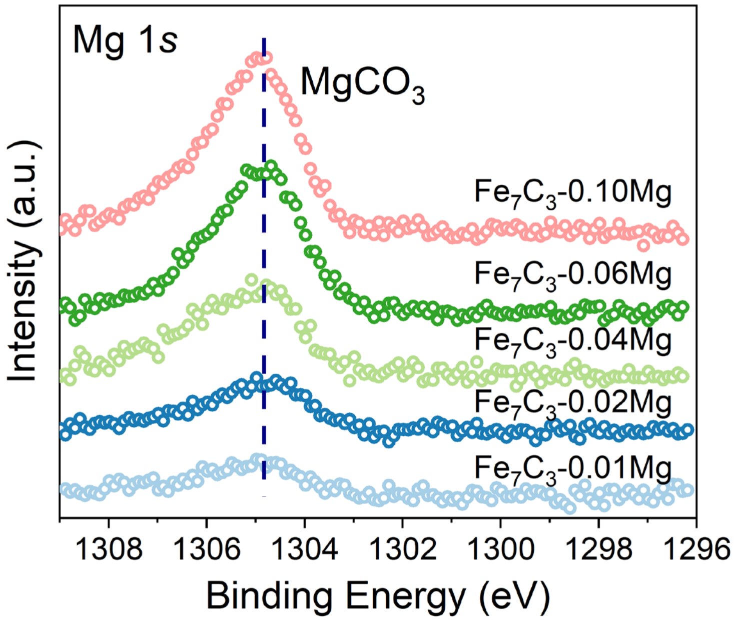

Supplementary Fig. 17 | Mg 1*s* XPS spectra of Fe_7C_3 catalysts with various addition magnesium levels.

The formation of $MgCO_3$ under CO_2-rich conditions (340 °C, 2 MPa, $H_2/CO_2 = 3$) is thermodynamically favorable and likely occurs readily on the catalyst surface. This can be attributed to the significantly lower standard Gibbs free energy of formation for $MgCO_3$ (–1028 kJ/mol) compared to MgO (–569 kJ/mol), suggesting that Mg^{2+} species derived from precursors such as $Mg(NO_3)_2$ or MgO are prone to surface carbonation in the presence of CO_2. This tendency is consistent with previous CO_2 capture studies, where MgO has been shown to readily convert into $MgCO_3$ even at low temperature. In our case, the presence of in situ generated CO_2 appears sufficient to drive this transformation, as evidenced by the appearance of carbonate-related features in the XPS spectra. The absence of detectable metallic Mg or MgO signals further supports the conclusion that surface Mg species are nearly fully carbonated under reaction conditions.

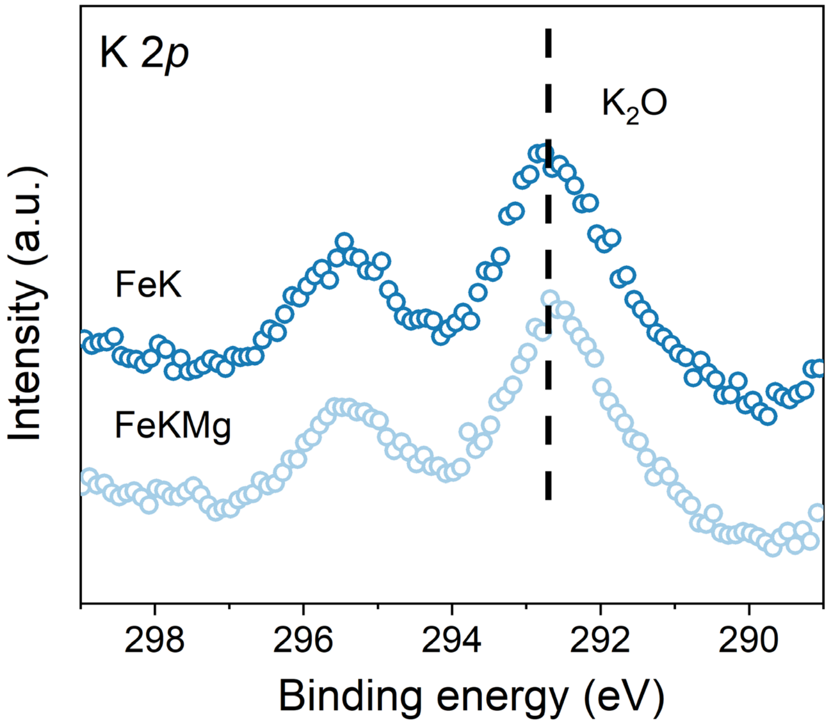

Supplementary Fig. 18 | K 2*p* XPS spectra of FeK and FeKMg catalysts.

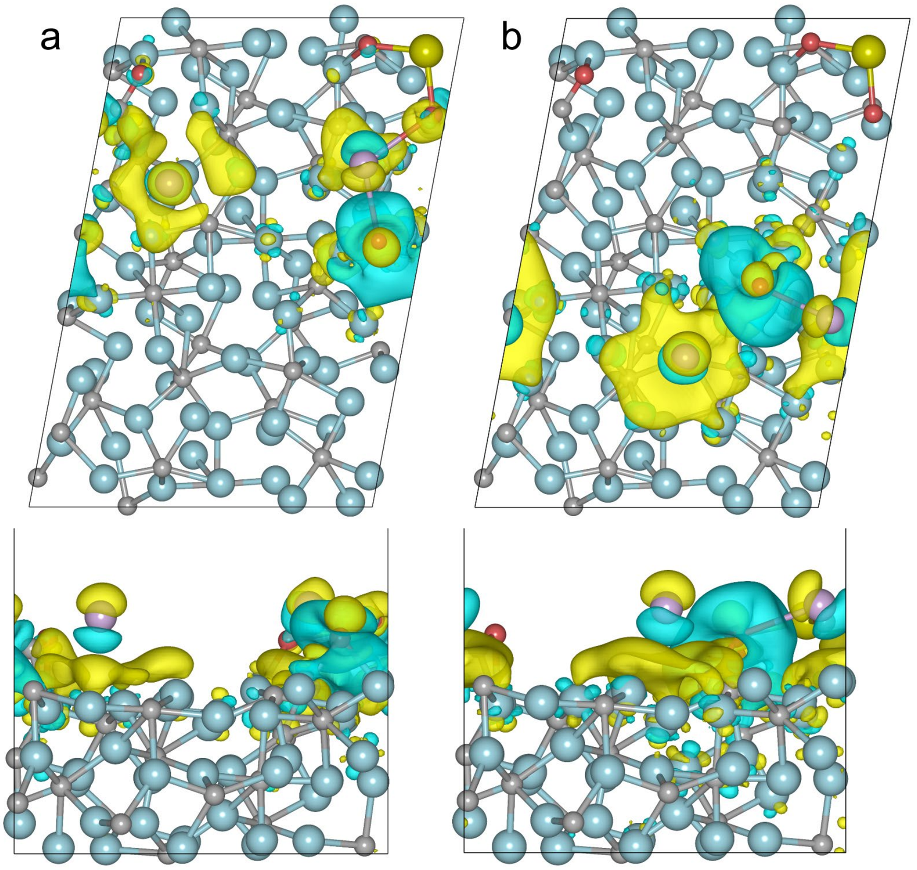

Supplementary Fig. 19 | The charge density difference for (a) Fe-KMg with K-Mg interaction, (b) Fe-KMg without K-Mg interaction. The yellow and blue regions represent electron accumulation and deficiency, respectively.

By calculating the charge density difference with and without K–Mg interactions, we quantified the extent of electronic coupling between the two promoters. Notably, no significant charge transfer between K and Mg was observed: in the interacting model, each K atom loses 0.839 e^-, compared to 0.806 e^- in the non-interacting model—a difference well within the margin of computational uncertainty. These results indicate minimal direct electronic interaction between K and Mg.

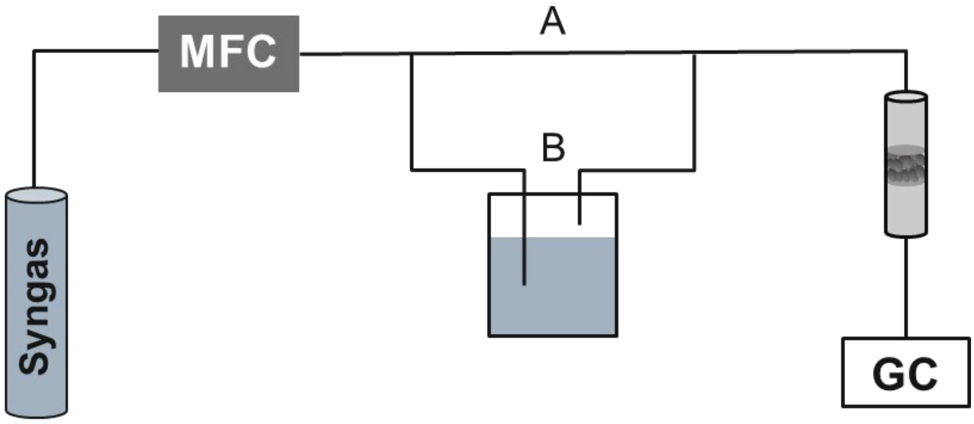

Supplementary Fig. 20 | Schematic diagram of bubbling water experimental device (A: No water added, B: Bubbling water).

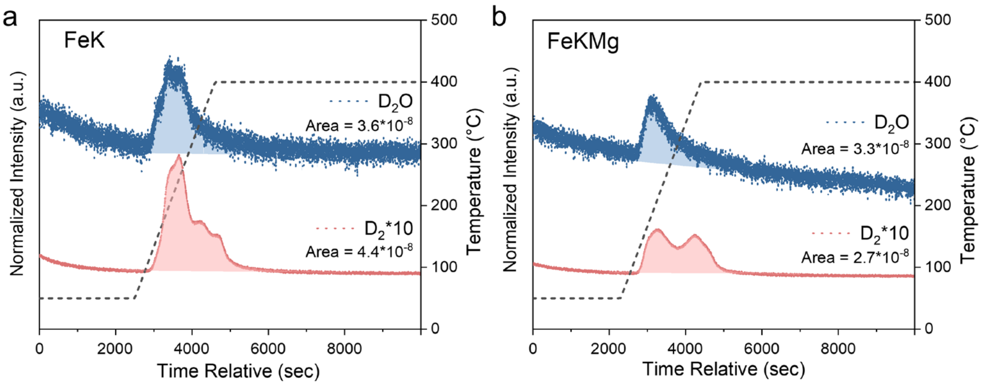

Supplementary Fig. 21 | (a) FeK, (b) FeKMg D_2O-TPD experiment

The D_2O-TPD experiment was conducted using the following procedure:

1. Weighed 100 mg activated catalyst sample and loaded it into a tube. The tube was installed in the apparatus, sealed.
2. Introduced Ar gas (total flow rate of 50 mL/min) and performed a programmed temperature increase to 300 °C (10 °C/min), holding the temperature until the mass spectrometry baseline stabilized.
3. Cooled the system to 50 °C and saturated the catalyst with D_2O adsorption for 20 minutes using Ar bubbling.
4. Switched to 95%Ar- 5%Kr flow and collected mass spectrometry signals (D_2O, D_2, Kr) for 30 minutes until the signals stabilized.
5. Increased the temperature to 400 °C under 95%Ar- 5%Kr atmosphere and maintained it for 30 minutes.

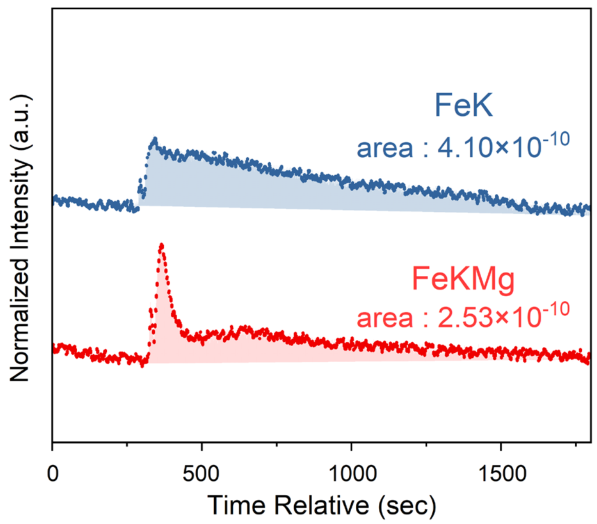

Supplementary Fig. 22 | Surface dissociated hydroxyl analysis experiment. (MS signal acquisition: D_2)

The following procedure was adopted for the D_2O transient switching experiment:

1. Weighed 100 mg activated catalyst sample and loaded it into a tube. The tube was installed in the apparatus, sealed.
2. Introduce Ar (total flow rate 50 mL/min) and program the temperature to ramp up to 300°C at a rate of 10 °C/min, holding it at that temperature until the mass spectrometry baseline stabilizes.
3. Use Ar to bubble and adsorb D_2O for 10 minutes.
4. Switched to Ar flow and collected mass spectrometry signals (Ar, D_2O) for 30 minutes until the signals stabilized.
4. Switch to a 5%CO-Ar mixture, and collect mass spectrometry signals for D_2.

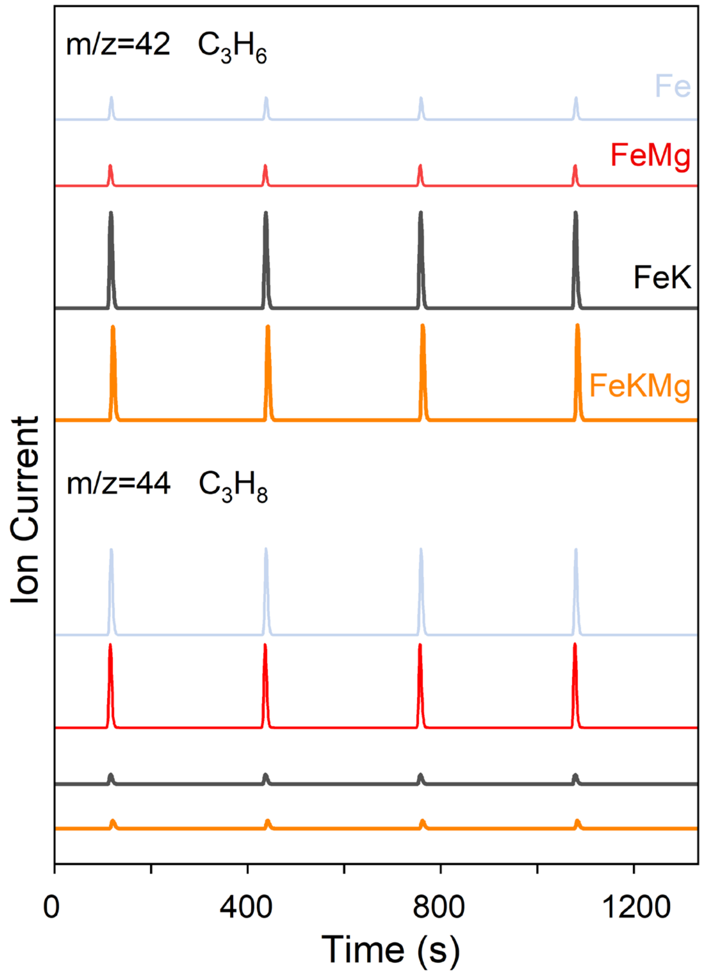

Supplementary Fig. 23 | C_3H_6 hydrogenation experiment. Catalysts were activated under ammonia gas, followed by a switch to 10% H_2/Ar (50 mL/min), with the temperature adjusted to 300 °C. C_3H_6 was then pulsed into the system to complete the PTH.

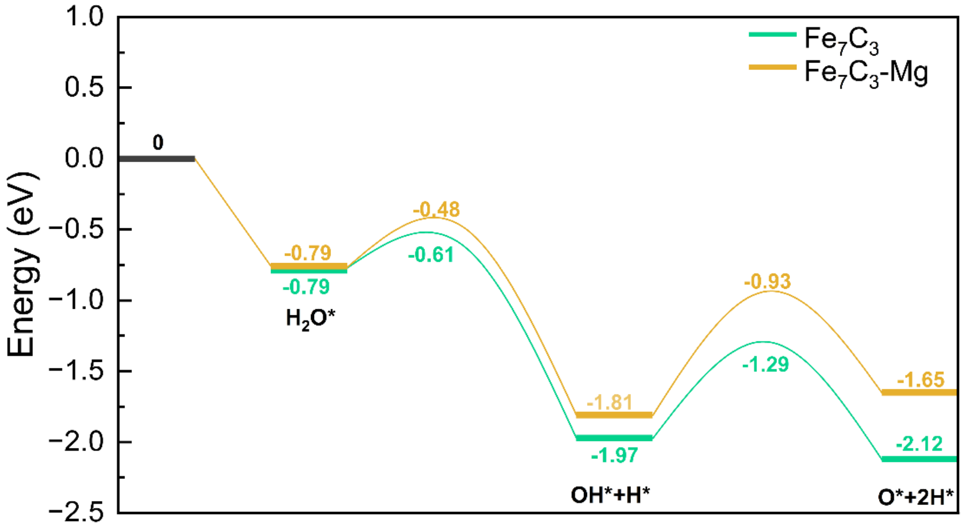

Supplementary Fig. 24 | The potential energy surface for H_2O dissociation on Fe_7C_3 and Fe_7C_3-Mg.

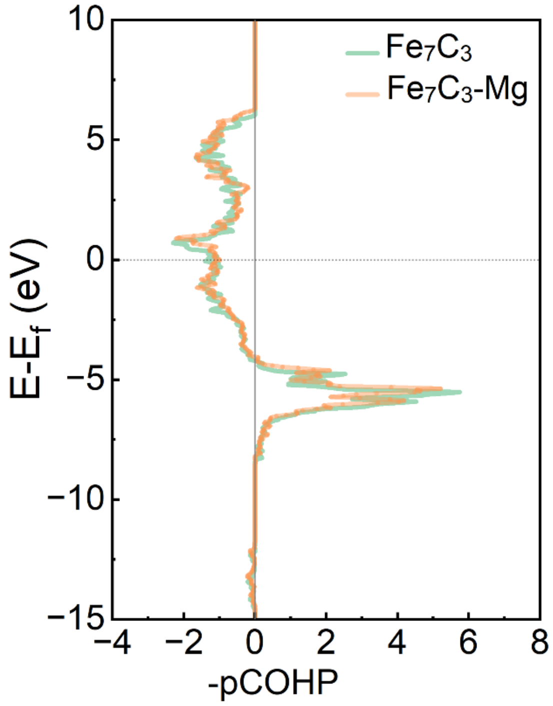

Supplementary Fig. 25 | The Crystal Orbital Hamilton Population for Fe-O bonds on Fe_7C_3 and Fe_7C_3-Mg.

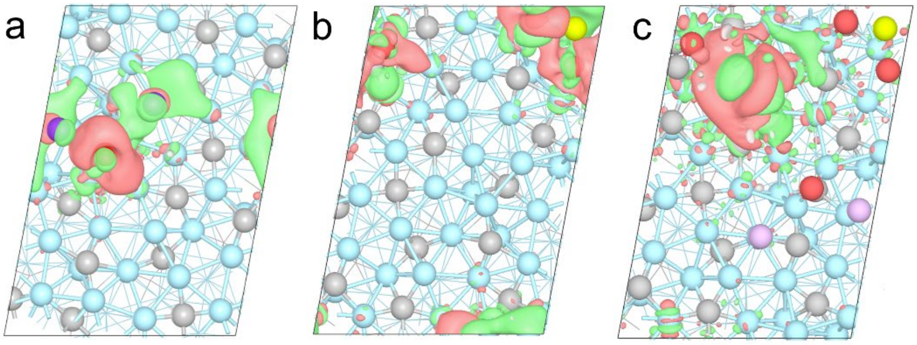

Supplementary Fig. 26 | The charge density difference between (a) K_2O and Fe_7C_3, (b) $MgCO_3$ and Fe_7C_3, (c) $K_2O/MgCO_3$ and Fe_7C_3, the green and red regions represent charge- accumulation and deficiency, respectively.

Supplementary Table 1| Fitting parameters of the Mössbauer spectra of spent catalyst. (corresponding to Fig. 1b, Supplementary Fig. 3)

Sample	IS ($mm{\cdot}s^{-1}$)	QS ($mm{\cdot}s^{-1}$)	Hhf (kOe)	Γ/2 ($mm{\cdot}s^{-1}$)	Area (%)	Assignment
Fe	0.40	0.01	51.62	0.50	39.61	Fe_3O_4 (A)
	0.70	-0.45	46.90	1.37	40.06	Fe_3O_4 (B)
	0.23	0.22	25.99	0.58	8.70	Fe_7C_3 (A)
	0.31	0.00	14.00	0.55	3.20	Fe_7C_3 (B)
	0.32	0.21	22.02	0.48	7.25	Fe_7C_3 (C)
	0.24	-0.57	-	0.29	1.19	Fe^{3+} (spm)
FeMg	0.40	-0.01	51.63	0.50	49.93	Fe_3O_4 (A)
	0.70	-0.48	46.90	1.18	24.01	Fe_3O_4 (B)
	0.25	0.25	25.69	0.5	13.79	Fe_7C_3 (A)
	0.22	0.03	22.00	0.55	7.03	Fe_7C_3 (B)
	0.40	-0.02	14.00	0.46	3.38	Fe_7C_3 (C)
	0.40	2.00	-	0.58	1.85	Fe^{3+} (spm)
FeK	0.40	-0.03	51.41	0.50	28.61	Fe_3O_4 (A)
	0.70	-0.70	46.90	1.07	16.91	Fe_3O_4 (B)
	0.32	0.13	25.28	0.49	21.95	Fe_7C_3 (A)
	0.27	0.00	21.16	0.58	18.93	Fe_7C_3 (B)
	0.28	-0.05	12.04	0.56	12.39	Fe_7C_3 (C)
	0.36	-1.07	-	0.29	1.20	Fe^{3+} (spm)
FeKMg	0.31	-0.03	24.74	0.53	41.64	Fe_7C_3 (A)
	0.26	0.16	18.70	0.52	43.36	Fe_7C_3 (B)
	0.30	-0.07	12.41	0.50	12.86	Fe_7C_3 (C)
	0.11	-0.99	-	0.29	2.15	Fe^{3+} (spm)

Supplementary Table 2 | Curve-fit Parameters [a] for Fe *K*-edge EXAFS of spent catalyst. (corresponding to Fig. 1d, Supplementary Fig. 4)

Catalyst	Path	d (Å) [b]	C. N. [c]	ΔE_0 (eV)	σ^2 (Å^2) [d]	R-factor
Fe [e]	Fe-C/O	1.97 ± 0.01	4.4 ± 0.5	-2 ± 1	0.014 ± 0.002	0.004
	$Fe\text{-}Fe_C$	2.51 ± 0.01	1.9 ± 0.5		0.008 ± 0.002	
	$Fe\text{-}Fe_O$	3.47 ± 0.01	13.3 ± 1.5		0.015 ± 0.001	
	Fe-O	3.52 ± 0.03	13.3 ± 1.5		0.026 ± 0.007	
FeMg [e]	Fe-C/O	2.00 ± 0.05	5.1 ± 1.0	-1 ± 3	0.014 ± 0.003	0.005
	$Fe\text{-}Fe_C$	2.53 ± 0.02	2.6 ± 1.0		0.010 ± 0.003	
	$Fe\text{-}Fe_O$	3.48 ± 0.05	13.4 ± 2.3		0.015 ± 0.002	
	Fe-O	3.53 ± 0.03	13.4 ± 2.3		0.022 ± 0.014	
FeK [e]	Fe-C/O	1.97 ± 0.05	3.9 ± 0.9	-2 ± 1	0.013 ± 0.003	0.009
	$Fe\text{-}Fe_C$	2.66 ± 0.02	8.2 ± 1.4		0.017 ± 0.002	
	$Fe\text{-}Fe_O$	3.58 ± 0.04	5.6 ± 1.5		0.023 ± 0.007	
	Fe-O	3.41 ± 0.02	5.6 ± 1.5		0.008 ± 0.005	
FeKMg [e]	Fe-C	1.97 ± 0.01	2.6 ± 0.3	-7 ± 1	0.007 ± 0.003	0.003
	$Fe\text{-}Fe_{C1}$	2.48 ± 0.02	2.6 ± 0.3		0.006 ± 0.002	
	$Fe\text{-}Fe_{C2}$	2.62 ± 0.01	7.8 ± 0.8		0.009 ± 0.002	
Fe_7C_3	Fe-C	2.05 ± 0.02	1.6 ± 0.6	1 ± 1	0.005 ± 0.004	0.003
	$Fe\text{-}Fe_{C1}$	2.51 ± 0.04	2.7 ± 0.6		0.013 ± 0.008	
	$Fe\text{-}Fe_{C2}$	2.65 ± 0.03	8.1 ± 1.8		0.012 ± 0.003	

a: The data ranges used in these fittings are 3.0 ⩽ k ⩽ 13.0 Å^{-1} and 1.0 ⩽ R ⩽ 3.0 Å (for FeKMg and Fe_7C_3), 1.0 ⩽ R ⩽ 3.5 Å (for others). S_0^2 was fixed at 0.68 or 0.74, obtained from the Fe foil measured at the same time. The number of variable parameters in each fitting is out of total independent data points.

b: The coordination distance. The distances for Fe-C, Fe-O and Fe-Fe are from the crystal structure of Fe_7C_3 (*P*6$_3$*mc*, ICSD collection code 76830) and Fe_3O_4 (*Fd*-3*mZ*, ICSD collection code 26410).

c: Path degeneracy, which is identical to the average coordination number of single scattering paths.

d: Debye-Waller factor.

e: To limit the number variables in the fit, we used the proportional relationship between the main paths in the Fe_3O_4 and Fe_7C_3 crystal structures to constrain the parameters: For FeKMg, C. N. (Fe-C) = C. N (Fe-Fe_{C1}) = 1/3 C. N. (Fe-Fe_{C2}); For Fe_7C_3, C. N (Fe-Fe_{C1}) = 1/3 C. N. (Fe-Fe_{C2}); For others, C. N. (Fe-C/O) = 1/3 C. N (Fe-Fe_O) +1/4 C. N. (Fe-Fe_C). Since the Fe-O path in Fe_3O_4 (1.94 Å) is extremely close to the Fe-C path in Fe_7C_3 (2.05 Å), we use only the Fe-O path instead of two paths.

Supplementary Table 3 | Elemental contents of different catalysts.

Catalysts	Element contents (wt%)		
	Fe	K	Mg
Fe	58	-	-
FeMg	57	-	-
FeK	53	8	-
FeKMg	52	8	3

Supplementary Table 4 | Fitting parameters of the Mössbauer spectra of spent catalyst in 8 h. (corresponding to Supplementary Fig. 8)

Sample	IS ($mm \cdot s^{-1}$)	QS ($mm \cdot s^{-1}$)	Hhf (kOe)	Γ/2 ($mm \cdot s^{-1}$)	Area (%)	Assignment
Fe	0.40	-0.01	51.39	0.50	40.81	Fe_3O_4 (A)
	0.70	-0.32	46.90	1.34	36.41	Fe_3O_4 (B)
	0.29	-0.33	12.49	0.29	2.49	Fe_7C_3 (A)
	0.27	0.03	21.52	0.57	9.99	Fe_7C_3 (B)
	0.28	0.15	25.20	0.52	9.36	Fe_7C_3 (C)
	0.00	-0.32	-	0.29	0.95	Fe^{3+} (spm)
FeMg	0.40	-0.02	51.27	0.50	46.09	Fe_3O_4 (A)
	0.70	-0.50	46.90	1.27	29.60	Fe_3O_4 (B)
	0.27	-0.33	12.31	0.23	2.16	Fe_7C_3 (A)
	0.29	0.00	21.25	0.58	12.86	Fe_7C_3 (B)
	0.28	0.04	25.41	0.39	8.31	Fe_7C_3 (C)
	0.40	1.29	-	0.29	0.98	Fe^{3+} (spm)
FeK	0.32	0.12	25.44	0.52	33.09	Fe_7C_3 (A)
	0.22	0.00	21.27	0.56	22.14	Fe_7C_3 (B)
	0.32	0.14	13.39	0.58	21.81	Fe_7C_3 (C)
	0.33	0.13	18.33	0.45	20.97	Fe_2C
	0.40	1.20	-	0.29	2.00	Fe^{3+} (spm)
FeKMg	0.32	-0.01	25.62	0.55	27.90	Fe_7C_3 (A)
	0.11	0.02	21.46	0.58	14.46	Fe_7C_3 (B)
	0.32	0.12	13.57	0.58	22.45	Fe_7C_3 (C)
	0.32	0.13	18.11	0.50	32.81	Fe_2C
	0.4	1.05	-	0.29	2.37	Fe^{3+} (spm)

Supplementary Table 5 | Catalytic properties of Fe_7C_3 with different magnesium additions level.

Catalysts	CO_2 Conversion [%]	C_1 sel.[mol%]			C_{2-4} sel. [mol%]	C_{5+}sel. [mol%]	C_{2-4} O/P	$C_{2+}^{=}$ sel. [mol%]
		CO	CH_4	Total				
Fe_7C_3-K	35.9	28.6	12.9	41.5	36.4	22.1	6.6	48.8
Fe_7C_3-K-0.01Mg	36.6	13.9	10.2	24.1	37.7	38.2	7.3	64.6
Fe_7C_3-K-0.02Mg	37.2	14.1	9.4	23.5	35.2	41.3	7.5	65.0
Fe_7C_3-K-0.04Mg	41.2	11.5	10.4	21.9	37.4	39.6	7.9	67.1
Fe_7C_3-K-0.06Mg	39.3	14.9	10.0	24.9	36.3	38.8	7.8	64.2
Fe_7C_3-K-0.10Mg	38.2	16.7	9.8	26.6	37.2	36.2	7.7	62.8

Reaction conditions: 0.10 g catalyst, 340 °C, 2.0 MPa, H_2/CO_2 = 3, GHSV = 6 $L \cdot g_{cat}^{-1} \cdot h^{-1}$.

Supplementary Table 6 | Fitting parameters of the Mössbauer spectra of spent catalyst with different Mg contents. (corresponding to Supplementary Fig. 12)

Sample	IS ($mm \cdot s^{-1}$)	QS ($mm \cdot s^{-1}$)	Hhf (kOe)	Γ/2 ($mm \cdot s^{-1}$)	Area (%)	Assignment
Fe_7C_3-K-0.01Mg	0.40	-1.73	-	0.29	2.36	Fe^{3+}(spm)
	0.31	-0.10	24.63	0.58	44.60	Fe_7C_3 (A)
	0.24	0.20	18.46	0.47	47.93	Fe_7C_3 (B)
	0.30	-0.08	12.75	0.20	5.12	Fe_7C_3 (C)
Fe_7C_3-K-0.01Mg	0.40	-1.64	-	0.29	1.39	Fe^{3+}(spm)
	0.31	0.01	24.53	0.58	47.23	Fe_7C_3 (A)
	0.25	0.20	18.86	0.57	39.09	Fe_7C_3 (B)
	0.30	-0.04	12.76	0.40	12.29	Fe_7C_3 (C)
Fe_7C_3-K-0.01Mg	0.11	-0.99	-	0.29	2.15	Fe^{3+}(spm)
	0.31	-0.03	24.74	0.53	41.64	Fe_7C_3 (A)
	0.26	0.16	18.70	0.52	43.36	Fe_7C_3 (B)
	0.30	-0.07	12.41	0.50	12.86	Fe_7C_3 (C)
Fe_7C_3-K-0.01Mg	0.19	-1.20	-	0.29	1.67	Fe^{3+}(spm)
	0.30	-0.01	24.55	0.58	47.26	Fe_7C_3 (A)
	0.22	0.20	18.95	0.56	39.31	Fe_7C_3 (B)
	0.23	0.06	12.55	0.40	11.76	Fe_7C_3 (C)
Fe_7C_3-K-0.01Mg	0.36	-1.57	-	0.29	2.31	Fe^{3+}(spm)
	0.32	-0.10	24.65	0.58	45.84	Fe_7C_3 (A)
	0.25	0.20	18.56	0.44	43.54	Fe_7C_3 (B)
	0.30	0.06	12.88	0.26	8.32	Fe_7C_3 (C)

Supplementary Table 7 | Catalytic properties of Fe_7C_3 with different temperature.

Catalysts	Temperature	CO_2	C_1 sel.[mol%]			C_{2-4} sel.	C_{5+}sel.	C_{2-4}	$C_{2+}^{=}$ sel.
	(°C)	Conversion [%]	CO	CH_4	Total	[mol%]	[mol%]	O/P	[mol%]
Fe_7C_3-K-0.04Mg	340	41.2	11.5	10.4	21.9	37.4	39.6	7.9	67.1
Fe_7C_3-K-0.04Mg	360	43.1	16.3	10.1	26.3	33.8	39.9	7.8	63.2
Fe_7C_3-K-0.04Mg	380	45.6	17.4	12.3	29.7	31.7	28.6	7.7	59.5
Fe_7C_3-K-0.04Mg	400	47.0	19.7	16.4	36.1	37.2	26.7	6.7	53.2

Reaction conditions: 0.10 g catalyst, 340 - 400 °C, 2.0 MPa, H_2/CO_2 = 3, GHSV = 6 $L \cdot g_{cat}^{-1} \cdot h^{-1}$.

Supplementary Table 8 | Catalytic properties of Fe_7C_3 with different gas hourly space velocity.

Catalysts	GHSV ($L \cdot g_{cat}^{-1} \cdot h^{-1}$)	CO_2 Conversion [%]	C_1 sel.[mol%]			C_{2-4} sel. [mol%]	C_{5+}sel. [mol%]	C_{2-4} O/P	$C_{2+}^{=}$ sel. [mol%]
			CO	CH_4	Total				
Fe_7C_3-K-0.04Mg	6	41.2	11.5	10.4	21.9	37.4	39.6	7.9	67.1
Fe_7C_3-K-0.04Mg	10	37.0	19.2	11.1	30.3	38.8	30.9	7.9	59.8
Fe_7C_3-K-0.04Mg	12	35.5	21.4	8.7	30.1	42.9	27.0	7.9	58.0

Reaction conditions: 0.10 g catalyst, 340 °C, 2.0 MPa, H_2/CO_2 = 3, GHSV = 6-12 $L \cdot g_{cat}^{-1} \cdot h^{-1}$.

Supplementary Table 9 | Comparison of the catalytic performance of Fe_7C_3 with CO_2 hydrogenation and other catalysts reported in the literature.

Entry	Catalysts	GHSV ($ml \cdot g_{cat}^{-1} \cdot h^{-1}$)	T (°C)	H_2/CO_2 ratio	CO_2 Conv [%]	Sel. [mol%] CO	CH_4	High value-added olefins	Ref.
1.	Fe/C-bio	2240	320	3	30.5	23.2	9.1	55.3	3
2.	NaFe	2000	320	3	40.5	13.5	13.7	63.2	4
3.	10Mn-Na/Fe	2040	320	3	37.7	12.9	14	29.2[a]	5
4.	Na-$CoFe_2O_4$	7200	320	3	41.8	9.7	20	33.6[a]	6
5.	10Mn-Fe_3O_4	4000	350	3	44.7	9.4	19.9	41.9[a]	7
6.	NaCoFe/CNT	3600	340	3	34.4	18.6	12.1	31.6	8
7.	1Fe-1Zn-K	1000	320	3	37.8	11.13	41.2	36.0	9
8.	Fe/Co-Y_k	2400	300	2.65	25.9	21.1	11.0	55.9	10
9.	Fe/C-K_2CO_3	2400	320	3	32.4	21.4	10.0	58.4	11
10.	1Na50Co50Fe	2000	270	2.5	22.7	42.1	35	<16.7	12
11.	ZnFeAlO4/SAPO	3600	380	6	48.5	48	-	41.6[a]	13
12.	K-Fe/MWNTs	9000	340	3	43.6	23.4	20.8	23.5[a]	14
13.	ZnZrOx/SSZ-13	4500	360	3	9.1	32	1.53	60.8	15
14.	Mn0.5Zn1.5Fe4O8	6000	310	3	26.6	20.2	28.1	25.1[a]	16
15.	$ZnGa_2O_4$/SAPO	5400	370	3	13	46	0.5	46.4	17
16.	KZnCo0.5Fe1.5O4	4800	320	3	49.6	5.8	17.8	34[a]	18
17.	In–Zr/SAPO-34	9000	400	3	35.5	85	0.7	12.8	19
18.	C/Co3@C/Fe	6000	320	3	33.4	23.8	11.0	29.6[a]	20
19.	FeCo-9:1-LDH	7200	320	2	40.6	9.6	18	32.9	21
20.	In_2O_3/SAPO-34	9000	350	3	14.1	60.9	0.7	30.1	22
21.	GaZrOx/SAPO-17	4800	375	3	9	62	0.9	31.4	23
22.	ZnZrO/SAPO	3600	380	3	12.6	47	1.6	43.5	24
23.	FeSrNa	8000	320	3	40.5	8.3	8.9	71.1	25
24.	Fe_7C_3-K-0.04Mg	6000	340	3	41.2	10.4	11.5	67.1	This work (a)
25.	Fe_7C_3-K-0.04Mg	12000	340	3	35.5	21.4	8.7	58.0	This work (b)

a: The values denote the selectivity of lower olefins ($C_{2-4}^{=}$)

Supplementary References

1. Gao, Z. et al. Shielding Pt/γ-Mo_2N by inert nano-overlays enables stable H_2 production. *Nature* **638**, 690-696 (2025).
2. Qian, F. et al. Stabilized ε-Fe_2C catalyst with Mn tuning to suppress C1 byproduct selectivity for high-temperature olefin synthesis. *Nat. Commun.*, **15**, 5128 (2024).
3. Guo, L. et al. Directly converting carbon dioxide to linear α-olefins on bio-promoted catalysts. *Commun. Chem.* **1**, 11 (2018).
4. Wei, J. et al. New insights into the effect of sodium on Fe_3O_4- based nanocatalysts for CO_2 hydrogenation to light olefins. *Catal. Sci. Technol.* **6**, 4786-4793 (2016).
5. Liang, B. et al. Mn decorated Na/Fe catalysts for CO_2 hydrogenation to light olefins. *Catal. Sci. Technol.* **9**, 456-464 (2019).
6. Yuan, F. et al. Boosting light olefin selectivity in CO_2 hydrogenation by adding Co to Fe catalysts within close proximity. *Catal. Today* **371**, 142-149 (2021).
7. Jiang, J. et al. Manganese-promoted Fe_3O_4 microsphere for efficient conversion of CO_2 to light olefins. *Ind. Eng. Chem. Res.* **59**, 2155-2162 (2020).
8. Kim, K. Y. et al. Cobalt ferrite Nanoparticles to form a catalytic Co-Fe alloy carbide phase for selective CO_2 hydrogenation to light olefins. *ACS Catal.* **10**, 8660-8671 (2020).
9. Zhang, J. et al. Selective formation of light olefins from CO_2 hydrogenation over Fe–Zn–K catalysts. *J. CO2 Util.* **12**, 95-100 (2015).
10. Guo, L. et al. Selective formation of linear-alpha olefins (LAOs) by CO_2 hydrogenation over bimetallic Fe/Co-Y catalyst. *Catal. Commun.* **130**, 105759 (2019).
11. Han, Y. et al. Interfacing with carbonaceous potassium promoters boosts catalytic CO_2 hydrogenation of iron. *ACS Catal.* **10**, 12098-12108 (2020).
12. Gnanamani, M. K. et al. Hydrogenation of carbon dioxide over Co–Fe bimetallic catalysts. *ACS Catal.* **6**, 913-927 (2016).
13. Wang, X. et al. Breaking the activity-selectivity trade-off of CO_2 hydrogenation to light olefins. *Proc Natl Acad Sci U S A* **121**, e2408297121 (2024).
14. Wang, S. et al. Iron-potassium on single-walled carbon nanotubes as efficient catalyst for CO_2 hydrogenation to heavy olefins. *ACS Catal.* **10**, 6389-6401 (2020).
15. Chen, S. et al. Hydrogenation of CO_2 to light olefins over ZnZrOx /SSZ-13. *Angew. Chem. Int. Ed.* **63**, e202316874 (2024).
16. Liu, Y., Kishimoto, F., Lu, X., Li, J., Takanabe, K. CO_2 hydrogenation over Fe-Mn-Zn spinel oxide nanohybrids precatalysts. *Appl. Catal. B* **361**, 124675 (2025).
17. Liu, X. et al. Selective transformation of carbon dioxide into lower olefins with a bifunctional catalyst composed of $ZnGa_2O_4$ and SAPO-34. *Chem. Comm.* **54**, 140-143 (2018).
18. Xu, Q., Xu, X., Fan, G., Yang, L., Li, F. Unveiling the roles of Fe-Co interactions over ternary spinel-type $ZnCo_xFe_{2-x}O_4$ catalysts for highly efficient CO_2 hydrogenation to produce light olefins. *J. Catal.* **400**, 355-366 (2021).
19. Gao, P. et al. Direct production of lower olefins from CO_2 conversion via bifunctional catalysis. *ACS Catal.* **8**, 571-578 (2017).
20. Wang, X. et al. Isolated dual-active Fe-Co sites efficiently promote CO_2 hydrogenation upgrading. *Fuel* **364**, 131054 (2024).
21. Yuan, F. et al. Boosting the production of light olefins from CO_2 hydrogenation over Fe-Co bimetallic catalysts derived from layered double hydroxide. *Ind. Eng. Chem. Res.* **62**, 8210-8221 (2023).

22. Lu, S. et al. Effect of In_2O_3 particle size on CO_2 hydrogenation to lower olefins over bifunctional catalysts. *Chin. J. Catal.* **42**, 2038-2048 (2021).
23. Qin, L. et al. Selective hydrogenation of CO_2 into ethene and propene over a GaZrOx/H-SAPO-17 composite catalyst. *ACS Catal.* **13**, 11919-11933 (2023).
24. Li, Z. et al. Highly selective conversion of carbon dioxide to lower olefins. *ACS Catal.* **7**, 8544-8548 (2017).
25. Orege, J. I. et al. Highly stable Sr and Na co-decorated Fe catalyst for high-valued olefin synthesis from CO_2 hydrogenation. *Appl. Catal. B* **316**, 121640 (2022).
